# Exploring anti-SARS-CoV-2 natural products: dual-viral target inhibition by delphinidin and the anti-coronaviral efficacy of deapio platycodin D

**DOI:** 10.1007/s13659-025-00523-w

**Published:** 2025-06-13

**Authors:** Jiani Lu, Yan Tang, Hongtao Li, Saisai Tian, Xixiang Chen, Xueyue Song, Pengcheng Qin, Jianrong Xu, Haiyan Zhu, Liqiang Ni, Huarong Du, Weidong Zhang, Weihua Li, Lili Chen

**Affiliations:** 1 The Research Center for Traditional Chinese Medicine, Shanghai Institute of Infectious Diseases and Biosecurity, Shanghai Frontiers Science Center of TCM Chemical Biology, Institute of Interdisciplinary Integrative Medicine Research, Shanghai University of Traditional Chinese Medicine, Shanghai, 201203 China; 2 Longhua Hospital, Shanghai University of Traditional Chinese Medicine, Shanghai, 201203 China; 3 Shanghai Frontiers Science Center of Optogenetic Techniques for Cell Metabolism, Shanghai Key Laboratory of New Drug Design, School of Pharmacy, East China University of Science and Technology, Shanghai, 200237 China; 4 School of Pharmacy, Second Military Medical University, Shanghai, 200433 China; 5 School of Pharmacy, Henan University, Kaifeng, 475001 China; 6 Department of Biological Medicines & Shanghai Engineering Research Center of Immunotherapeutics, School of Pharmacy, Fudan University, Shanghai, 201203 China; 7 Shanghai University of Traditional Chinese Medicine, Shanghai, 201203 China

**Keywords:** Qingfei Paidu decoction (QFPDD), SARS-CoV-2, S-RBD, Delphinidin, Deapio platycodin D

## Abstract

Qingfei Paidu decoction (QFPDD) has been extensively used in clinical treatments during the severe acute respiratory syndrome coronavirus 2 (SARS-CoV-2) epidemic. SARS-CoV-2 primarily invades host cells via its spike (S) protein binding to the angiotensin-converting enzyme 2 (ACE2) on the cell membrane, mediating viral-host membrane fusion. Blocking viral entry is a crucial step in preventing infection, with the interaction between the S receptor binding domain (S-RBD) and ACE2 being a key antiviral target. Given that SARS-CoV-2 predominantly affects the respiratory system and approximately 25% of patients suffering from corona virus disease 2019 (COVID-19) with gastrointestinal symptoms, we are committed to identifying more active ingredients in QFPDD that target the respiratory and gastrointestinal tracts of COVID-19 patients. Among medicinal plants, ephedra and liquorice derived from QFPDD, along with two other Chinese herbs, *Platycodon grandiflorum* and *Radix Rhei Et Rhizome* (rhubarb), have garnered our interest. These herbs have historically been used in traditional Chinese medicine (TCM) for treating infectious diseases with respiratory and digestive symptoms. Here, we established a library containing all components of the four individual herbs gathered from the Traditional Chinese Medicine Systems Pharmacology Database and Analysis Platform (TCMSP) and performed structure-based virtual screening to identify potential ACE2/S-RBD inhibitors. Subsequently, we selected 10 ingredients from the top 30 candidates and evaluated their activities using a pseudovirus neutralization assay. Delphinidin and deapio platycodin D (DPD) showed significant antiviral potential with half-maximal inhibitory concentration (IC_50_) values of 45.35 µM and 1.38 µM, respectively. Furthermore, delphinidin also inhibited the 3-chymotrypsin-like protease (3CL^pro^), indicating its dual-viral target inhibitory potential. Notably, DPD effectively suppressed HCoV-229E replication in BEL-7402 cells. This study not only provides a strategy for rapid identifying antiviral agents from TCM in anticipation of future pandemics but also offers theoretical and experimental evidence to support for the clinical use of QFPDD.

**Graphical Abstract:**

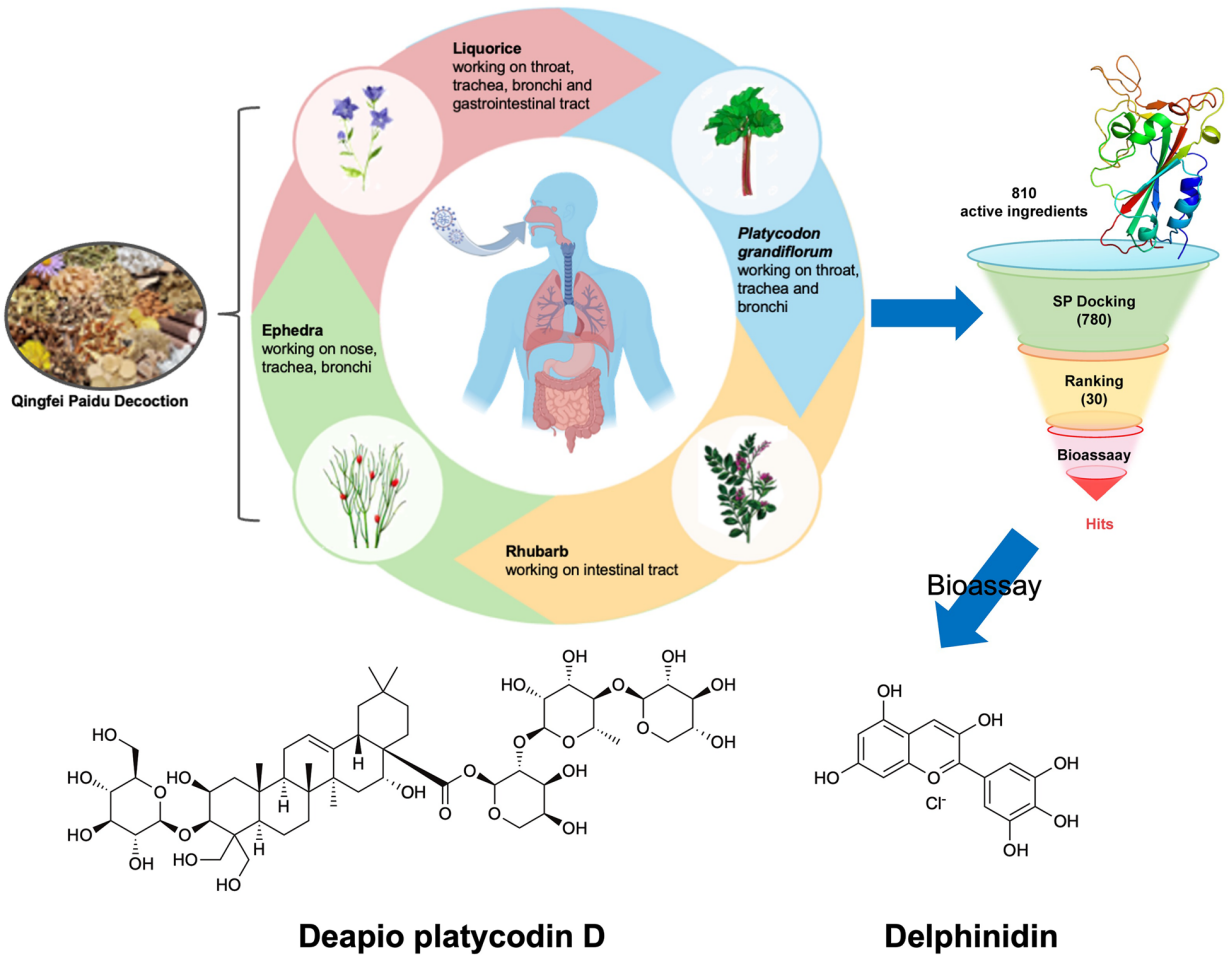

**Supplementary Information:**

The online version contains supplementary material available at 10.1007/s13659-025-00523-w.

## Introduction

Qingfei Paidu decoction (QFPDD), a well-known traditional Chinese medicine prescription for treating corona virus disease 2019 (COVID-19), has been applied to the diseases caused by the cold and damp invasion of the virus [1]. QFPDD combines four classic prescriptions—Maxing Shigan, Xiaochaihu, Shegan Mahuang decoctions and Wuling powder—historically used for cold, fever, and flu. The herbal ingredients of QFPDD, including their Latin names, are summarized in supplementary Table 1.

Extensive clinical investigations have shown that QFPDD, either alone or combined with conventional medicine, effectively improves patient recovery, reduces mortality, and alleviates clinical symptoms [2–4]. Research has identified several components in QFPDD with anti-the severe acute respiratory syndrome coronavirus 2 (anti-SARS-CoV-2) and anti-inflammatory properties, and demonstrated its immunomodulatory and organ-protective effects through various experimental and analytical methods [5]. Despite these advancements, the precise mechanisms underlying the amelioration of respiratory and gastrointestinal symptoms in COVID-19 patients by QFPDD, as well as the specific antiviral targets engaged by its active constituents, remain to be fully elucidated and warrant further investigation.

**Fig. 1:**
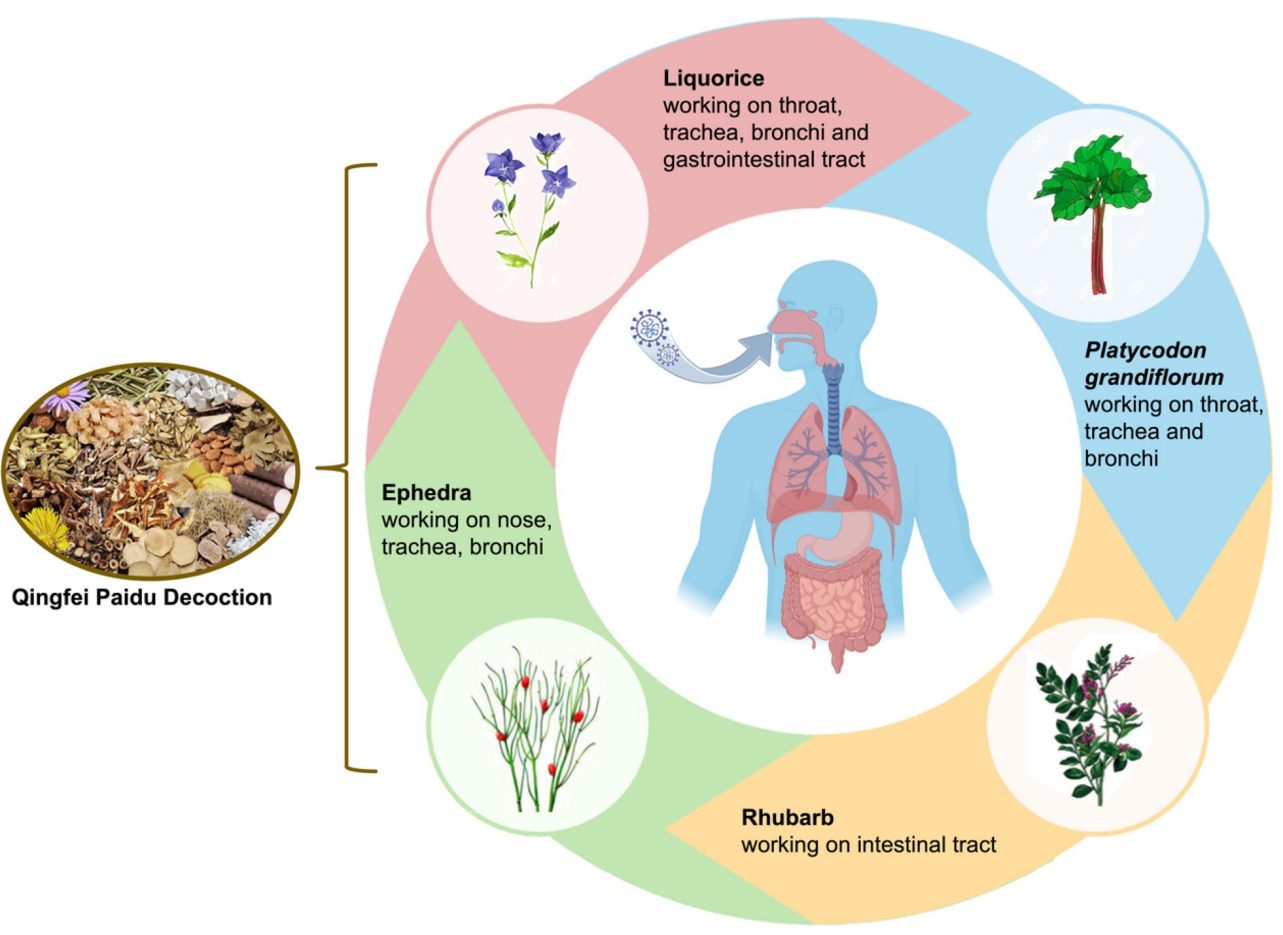
The main working sites of ephedra, liquorice, *Platycodon grandiflorum* and rhubarb in the body. Each Chinese herbal medicine has its unique properties, which are believed to have specific sites and effects on the human body in TCM theory

Since SARS-CoV-2 primarily infects the respiratory system and nearly 25% of COVID-19 patients also have gastrointestinal manifestations [6], we intend to devote a continuous endeavor for more active ingredients in QFPDD acting on the respiratory and gastrointestinal tract of COVID-19 patients. Among medicinal plants, ephedra and liquorice derived from QFPDD, and two other Chinese herbs (*Platycodon grandiflorum* and *Radix Rhei Et Rhizome* (rhubarb)) provoked our interest, because these herbs were first recorded in *Shennong’s Herbal Classic*, the first book of pharmacology in China, and were widely applyed to treating infectious diseases with respiratory and digestive system symptoms in the past. In traditional Chinese medicine (TCM) prescription theory, they are called “monarch drug”, which play major roles in the treatment of the underlying syndrome in these empirical compound prescriptions. Ephedra primarily affects the nasal passages, trachea, and bronchial passages [7, 8], whereas *Platycodon grandiflorum* mainly targets the throat, trachea, and bronchial passages [9, 10]. Rhubarb’s primary action is in the intestinal tract [11, 12]. Except for the throat, trachea and bronchi, liquorice is known for its efficacy in the gastrointestinal tract (Fig. 1) [13, 14]. These organs are also the primary sites of SARS-CoV-2 infection [15, 16]. Starting with the classical *Treatise on Febrile and Miscellaneous Diseases*, considered the precursor of TCM prescriptions, and moving on to *A Handbook of Prescriptions for Emergencies* which detailed antimalarial properties of artemisia annua, continuing to the *Treatise on Pestilence* from the Ming Dynasty and the *Differentiation of Warm Diseases* from the Qing Dynasty, these historical texts documented the medicinal properties of aforesaid four herbs [17]. Currently, research has also delved into the antiviral properties of these four herbs through laboratory experiments. Ephedra extracts devoid of ephedrine alkaloids inhibit SARS-CoV-2 replication [18]. Quinoline-2-carboxylic acid, pseudoephedrine (MHJ-17) and its derivative (MHJ-11) contained in ephedra are being explored as promising therapeutic agents for COVID-19 [19, 20]. Glycyrrhizin A3 (A3), glycyrrhetinic acid (GA), glycyrrhizic acid (ZZY-44), and schaftoside exhibit inhibition of SARS-CoV-2 infection and present potencial for COVID-19 prevention and therapy [21–23]. *Platycodon grandiflorum* has shown efficacy in inhibiting SARS-CoV-2 infection, with platycodon D identified as an effective natural product [24]. Additionally, emodin, a significant constituent of rhubarb, has demonstrated the capability to impede spike protein receptor binding domain (S-RBD) binding to angiotensin-converting enzyme 2 (ACE2) and 3-chymotrypsin-like protease (3CL^pro^) [25, 26]. Therefore, we aimed to find the active ingredients from the four herbs and provide a scientific basis for the TCM theory to prevent epidemics and eliminate diseases.

SARS-CoV-2 invades host cells by its spike (S) protein binding to the ACE2, primarily expressed in respiratory and digestive tract epithelial cells [27]. Blocking viral entry, particularly through the interaction of S and ACE2, is a key antiviral strategy. Using pseudoviruses as a lower-risk model for screening viral entry inhibitors, we identified small molecules targeting the S-ACE2 interaction [28–30].

Here, to discover the new ingredients against SARS-CoV-2 invasion more efficiently, we through computer-based virtual screening of a natural product library containing compounds from the four mentioned herbs, identified two components—delphinidin and deapio platycodin D (DPD). At non-cytotoxic doses, they exhibited effective inhibitory activity against the entry of pseudovirus enveloped with SARS-CoV-2 S protein into cells exogenously expressing ACE2 protein. Simultaneously, based on the combination of molecular simulations and biological testing, we discovered that delphinidin and DPD may exert their pseudovirus inhibition activity by blocking the binding of ACE2 and S-RBD. Moreover, delphinidin exhibits inhibitory activity against 3CL^pro^, indicating its potential as a dual-viral target agent. Due to the lack of the P3 condition, we determined the inhibitory activity of delphinidin and DPD against the alphacoronavirus HCoV-229E. Notably, DPD inhibits HCoV-229E replication in BEL-7402 cells with a half-maximal inhibitory concentration (IC_50_) of 3.30 µM. Consequently, our research deepens the understanding of QFPDD’s active components, laying a scientific foundation for prospective clinical applications in viral infections.

## Methods

### Materials

Sennoside A, sennoside B, DPD, protocatechuic acid, sennoside C, and D-glucuronic acid were purchased from Shanghai yuanye Bio-Technology Co., Ltd (Shanghai, China). Delanzomib, niclosamide, hesperidin, luteolin 7-O-glucuronide, gallic acid, delphinidin, GC376, and vitamin K3 were obtained from MedChemExpress (Monmouth Junction, USA). The chromatograms of the active ingredients delphinidin and DPD are provided in the supplementary materials.

Granules were obtained from the Department of Traditional Medicine, Shuguang Hospital, Shanghai University of Traditional Chinese Medicine. To prepare the stock solution, 100 mg of each granule was accurately weighed and transferred into a volumetric flask. One milliliter of ultrapure water was added to the granules, followed by vigorous shaking to facilitate dissolution. The mixture was then subjected to ultrasonication for 1 to 2 h using an ultrasonic processor (frequency: 35 kHz, power: 200 W) to ensure complete dissolution. Subsequently, the mixture was centrifuged at 15,000 rpm for 10 min at 4 °C to separate the supernatant. The collected supernatant was used as the stock solution with a concentration of 100 mg/mL.

### Cell culture and viability assay

HEK293T cells were sourced from ATCC (Manassas, Virginia, USA), while Vero-E6 cells, originating from African green monkey kidney epithelial tissue, were acquired from the Cell Bank of the Chinese Academy of Sciences (Shanghai, China). Both cell lines were cultured in Dulbecco’s modified Eagle’s medium (DMEM), supplemented with 10% fetal bovine serum (FBS) (Yeasen Biotechnology Co., Ltd., Shanghai, China), 100 U/mL penicillin, and 100 µg/mL streptomycin, and maintained at 37 ℃ in a humidified environment with 5% CO_2_.

HEK293F cells, also provided by the Cell Bank of the Chinese Academy of Sciences, were grown in OPM-293 CD05 medium (OPM Biosciences, Shanghai, China) under shaking conditions (125 rpm) at 37 ℃ with 8% CO_2_.

Cytotoxicity in HEK293T cells stably expressing hACE2 (hACE2-HEK293T) was assessed using the Cell Counting Kit-8 (CCK-8) from Meilunbio (Dalian, China). A 50 µL suspension of cells was plated in 96-well plates and incubated for 12 h. After pretreatment with 10 µL of a 10 × solution of the test compound at 37 ℃ for 1 h, 40 µL of fresh medium was added. The cells were incubated for another 24 h, after which the medium was refreshed and incubation continued for an additional 24 h. Following this, the medium was replaced with a basic medium containing 10% CCK-8 reagent, as per the manufacturer’s protocol.

Vero-E6 cells were seeded into 96-well plates at a density of 2 × 10^4^ cells per well and cultured for 8 h at 37 ℃. The test compounds, diluted in DMEM with 2% FBS, were added, and the cells were incubated for 24 h. Subsequently, the medium was exchanged with DMEM containing 10% CCK-8 solution, and the cells were incubated for 2 h. The optical density (OD) at 450 nm was measured using a BioTek microplate reader (Winooski, USA).

### Production of SARS-CoV-2 pseudovirus

SARS-CoV-2 pseudovirus was produced following a previously established method [29, 30]. Briefly, HEK293T cells were co-transfected with pNL4.3-Luc-R.E and an optimized SARS-CoV-2 S protein sequence provided by Prof. Lu Lu from Fudan University using the LipoFiter 3.0 transfection reagent (Hanheng Bio, Shanghai, China). The total transfection amount was 15 µg, maintaining a 1:4 ratio between the envelope and skelemin protein plasmids. Additionally, codon-optimized expression plasmids for SARS-CoV-2 Omicron (GenScript, Nanjing, China) and plasmids expressing Delta (#172320) and Gamma (#170450) spike proteins (Addgene, Watertown, USA) were similarly transfected. Six hours post-transfection, the medium was replaced with fresh DMEM. Two days later, the pseudovirus-containing supernatant was collected, filtered through a 0.45 µm membrane, and stored at − 80 ℃ in 1 mL aliquots.

### Pseudovirus neutralization assay

To assess the inhibition of pseudovirus binding by the compounds, we followed a previously reported method [29, 30]. hACE2-HEK293T cells were seeded at 2.5 × 10^5^ cells per well in 96-well plates (50 µL/well) and cultured for 24 h. Compounds were pre-diluted to 10 × concentrations, and 10 µL of each was added to the wells. After a 1-h incubation, 40 µL of pseudovirus was added, and the mixture was incubated at 37 ℃ for 24 h. Luciferase activity was measured using a luciferase assay kit (Meilunbio, Dalian, China), with luminescence detected by Cytation 5 (BioTek, Winooski, USA). IC_50_ values were calculated using nonlinear regression analysis in GraphPad Prism 8.0 (California, USA).

### Ligand database preparation

A total of 837 active ingredients from ephedra, liquorice, rhubarb and *Platycodon grandiflorum* were obtained from TCMSP [31] (https://tcmsp-e.com/tcmsp.php). After removing duplicate components and checking the charge states, 810 candidate compounds were retained. The initial three-dimensional structures were used for the virtual screening.

The crystal structure of ACE2 in complex with the RBD of SARS-CoV-2 S protein (PDB code: 6M0J) was chosen as the receptor structure for virtual screening. The protein was prepared with Protein Preparation Wizard in Maestro [32], with a pH of 7.0 ± 2.0, and the water molecules and other solvent molecules were removed. The bond orders were assigned and the hydrogens were appended. Other parameters were set as the default. Then, the hydrogen bond network of the protein was optimized and the protein structure was further energy-minimized by the OPLS4 force field. Finally, chain E of 6M0J, i.e., the S-RBD region ranging from T333 to G526 was extracted, which was used as the receptor for subsequent virtual screening. The crystal structure of SARS-CoV-2 3CL^pro^ (PDB code: 7SI9) was chosen to be the receptor for exploring the interactions between delphinidin and DPD with 3CL^pro^. The protein was prepared with the same procedure as the above SARS-CoV-2 S-RBD.

### Docking-based virtual screening

The docking-based virtual screening was accomplished by Glide [32]. In our previous study, R403 and Y505 of S-RBD were demonstrated to play an important role in the specific recognition of ACE2 [29]. Meanwhile, Q493 and Y449 are important residues that bind to ACE2 and form two hydrogen bonds with ACE2, respectively [33]. Therefore, the four residues R403, Y449, Q493 and Y505 were taken as the grid center. The grid box was set to 30 Å × 30 Å × 30 Å. The SP [34] mode of Glide was used to dock the compound library into the S-RBD binding site. The docking scoring function was used for ranking all outputs. Finally, the top 30 docking results were retained for further analysis and processing.

For 3CL^pro^, the center of the grid box was defined as the centroid of the co-crystallized ligand in 7SI9 (x = − 10.525, y = 39.901, z = − 18.324), and the size of the grid box was set to be similar to the ligand. Docking was performed using standard precision (SP), and docking poses were ranked based on the docking scoring function. The pose with the best docking score was selected for further analysis.

### Protein expression and purification

Plasmids encoding recombinant SARS-CoV-2 S-RBD with a His tag (His-S-RBD), generously provided by Prof. Chunhe Wang from the Shanghai Institute of Materia Medica, were transfected into HEK293F cells using PEI (Polysciences, Warrington, USA) as described previously [29, 30]. After five days of culturing, Ni–NTA resin (Smart-Lifesciences, Changzhou, China) was used to purify the His-S-RBD protein. The purified product was then analyzed by SDS-PAGE to confirm its purity and molecular weight.

### Flow cytometry

A mixture of 25 µL of 2 µM His-S-RBD and 25 µL of each compound at the required concentration was preincubated for 1 h before incubating with 50 µL of Vero-E6 cells at a density of 3 × 10^6^ for 1 h at room temperature. The cells were washed twice with PBS, followed by the addition of 100 µL CoraLite® Plus 647-conjugated anti-His-tag monoclonal antibody (Proteintech, Wuhan, China) at a 1:400 dilution and incubated at 4 °C for 30 min. Flow cytometry analysis was performed after two PBS washes (Beckman, California, USA).

### Surface plasmon resonance assay (SPR)

Binding affinities between compounds and ACE2 or S-RBD were evaluated using a Biacore T200 instrument (Cytiva, UK). ACE2 or S-RBD proteins were immobilized on a CM5 chip via amine-coupling chemistry, with ACE2 at pH 4.0  and S-RBD at pH 5.5. A mixture of 50 mM NHS and 200 mM EDC was injected to activate the surface, after which 20 µg/mL ACE2 was immobilized to achieve 15,020 RU, and 30 µg/mL S-RBD reached 7193 RU. Surface blocking was performed using 1 M ethanolamine at pH 8.5. Delphinidin with 5% DMSO was injected into the flow system and analyzed for 120 s, followed by 300 s of dissociation. Glycine–HCl at pH 3.0 was used for surface regeneration between analyte injections. Data were processed using double reference subtraction and solvent corrections. Binding affinities were determined using a Langmuir 1:1 binding model in Biacore Evaluation software (Cytiva, UK).

### Enzymatic inhibition assay

SARS-CoV-2 3CL^pro^ was expressed and purified following previously established methods [35–37]. For enzyme inhibition assays, recombinant 3CL^pro^ of SARS-CoV-2 and SARS-CoV (final concentration: 120 nM) was incubated with test compounds in 0.1 M PBS (pH 7.4) containing 1 mM EDTA for 30 min. The reaction was initiated with the addition of a 20 µM FRET-based peptide substrate (Dabcyl-KNSTLQSGLRKE-Edans) (GenScript, Nanjing, China), and fluorescence was measured after 20 min using a Cytation 5 plate reader (BioTek, Winooski, USA), with excitation at 340 nm and emission at 490 nm.

### Host protease inhibitory activity assay

The activities of cathepsin B (CTSB), cathepsin L (CTSL), and chymotrypsin C (CTRC) were assessed following established methods [35, 36]. CTSB (Sino Biological, Beijing, China) was diluted to 100 nM in sodium acetate buffer (pH 5.5) containing EDTA and DTT, then activated for 30 min at 30 °C. After further dilution to 500 pM, CTSB was incubated with the test compounds for 30 min, followed by the addition of 5 µM Z-Phe-Arg-AMC (NJPeptide Biotech, Nanjing, China) to initiate the reaction. Fluorescence was recorded using a Cytation 5 reader at excitation/emission wavelengths of 360/460 nm.

Similarly, CTSL (Sino Biological, Beijing, China) was diluted, activated, and incubated with the test compounds before Z-Phe-Arg-AMC was added, with fluorescence measurements taken under the same conditions. For CTRC (Novoprotein, Suzhou, China), a 5 µg/mL solution was incubated with the test compounds in Tris buffer, then activated with trypsin (Sigma, Saint Louis, USA). The reaction was initiated using 100 µM Succinyl-Ala-Ala-Pro-Phe-p-nitroanilide (NJPeptide Biotech, Nanjing, China), and absorbance was measured at 405 nm.

### Anti HCoV-229E activity determination in vitro

#### Cell culture and determination of maximum non-toxic concentration

BEL-7402 cells were initially seeded into culture vessels at an optimal density and incubated for 24 h at 37 °C in a humidified atmosphere containing 5% CO_2_ to ensure robust growth and complete adherence. Subsequently, the cells were treated with varying concentrations of the test compound. The cytopathic effect (CPE) was continuously monitored to determine the maximum non-toxic concentration (MNTC) of the compound for BEL-7402 cells, which is a crucial parameter for the safe conduct of subsequent experiments.

#### Viral infection and antiviral activity assay

After establishing the MNTC, the cells were infected with HCoV-229E virus. The infected cells were then treated with sample solutions diluted to concentrations below the MNTC to evaluate the antiviral activity of the sample solutions at different concentrations. The characteristic morphological changes indicative of HCoV-229E infection (CPE) were closely monitored daily under a light microscope. To quantify the extent of CPE, direct imaging and specialized image analysis software were employed. Specifically, the percentage of cells exhibiting CPE was determined by counting the affected cells under a microscope. The collected data were analyzed to calculate the proportion of cells displaying characteristic morphological changes of viral infection, thereby providing a reliable and objective basis for assessing antiviral efficacy.

#### RNA extraction and quantitative PCR

To facilitate downstream molecular analyses, total RNA was extracted from treated cells 24 h post-treatment. RNA extraction was performed using kits from Shandong Sparkjade Biotechnology Co., Ltd. (Jinan, China) according to the manufacturer's protocol. Briefly, cells were lysed in Trizol reagent, followed by phase separation with chloroform and precipitation with isopropanol. The RNA was washed with ethanol and dissolved in RNase-free water. Reverse transcription and quantitative PCR (qPCR) were performed using kits from Beyotime Biotechnology (Shanghai, China) and Abclonal (Wuhan, China) respectively. GAPDH was used as the reference gene. Primer sequences are detailed in Table 1.

**Table 1:** Primer information

Primer name	Sequence (5’-3’)
GAPDH	Forward GGAGCGAGATCCCTCCAAAAT Reverse GGCTGTTGTCATACTTCTCATGG
HCoV-229E	Forward TGGCCCCATTAAAAATGTGT Reverse CCTGAACACCTGAAGCCAAT

## Results

### SARS-CoV-2-S pseudovirus-based assay to measure the anti-pseudovirus activities of TCM and virtual screening strategy

**Fig. 2:**
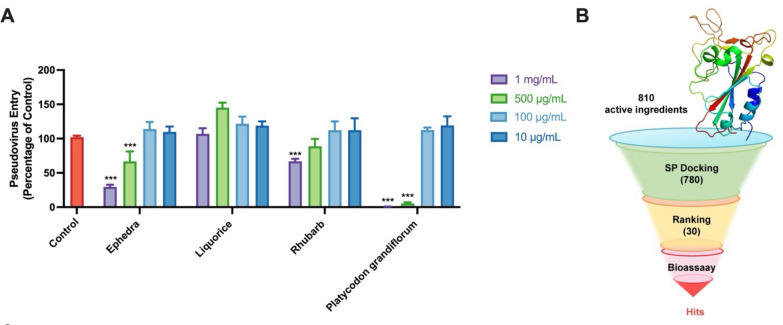
Pseudovirus activity test of herbs and virtual screening strategy. **A** The percentage of pseudovirus entering hACE2-HEK293T cells after treatment with aqueous solution of ephedra, liquorice, rhubarb and *Platycodon grandiflorum* granules at different concentrations. **B** The process of structure-based virtual screening is summarized in the flowchart, and the biological activity of the top 30 compounds with the highest molecular docking scores was validated through bioassays. Data are expressed as mean ± standard deviation (SD), and statistical significance was determined using P-values (***p < 0.001)

As mentioned above, we selected ephedra, liquorice, *Platycodon grandiflorum* and rhubarb based on their extensive use in the history of TCM for the treatment of infectious diseases. To test the antiviral activity of these four herbs, pseudovirus neutralization assay was applied to detect their inhibitory activity against SARS-CoV-2-S pseudovirus. As shown in Fig. 2A, ephedra, *Platycodon grandiflorum*, and rhubarb demonstrated the capacity to inhibit the virus from infiltrating hACE2-HEK293T cells. Based on our previous work, it was found that glycyrrhizin in liquorice might exhibit anti-SARS-CoV-2 activity by inhibiting the interaction between S-RBD and ACE2 [33]. Due to glycyrrhizin as the main active component of liquorice [38], liquorice was also selected for the subsequent virtual screening.

As depicted in Fig. 2B, 30 molecules of the 810 ingredients composed of the components of four herbs that were collected by TCMSP failed to be docked with the protein model. Thus, a total of 780 compounds were finally successfully docked. The top 30 compounds based on their molecular docking scores are listed in Table 2, which were further evaluated for biological activity.

**Table 2:**
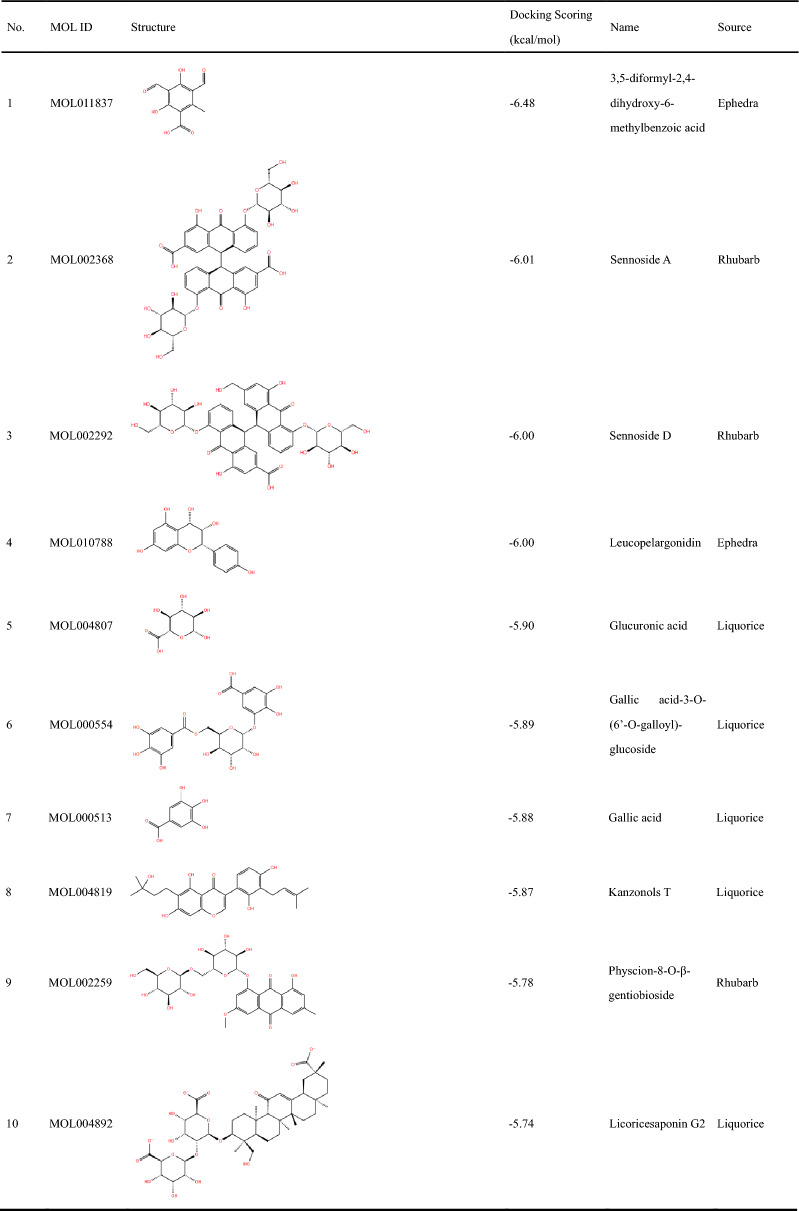

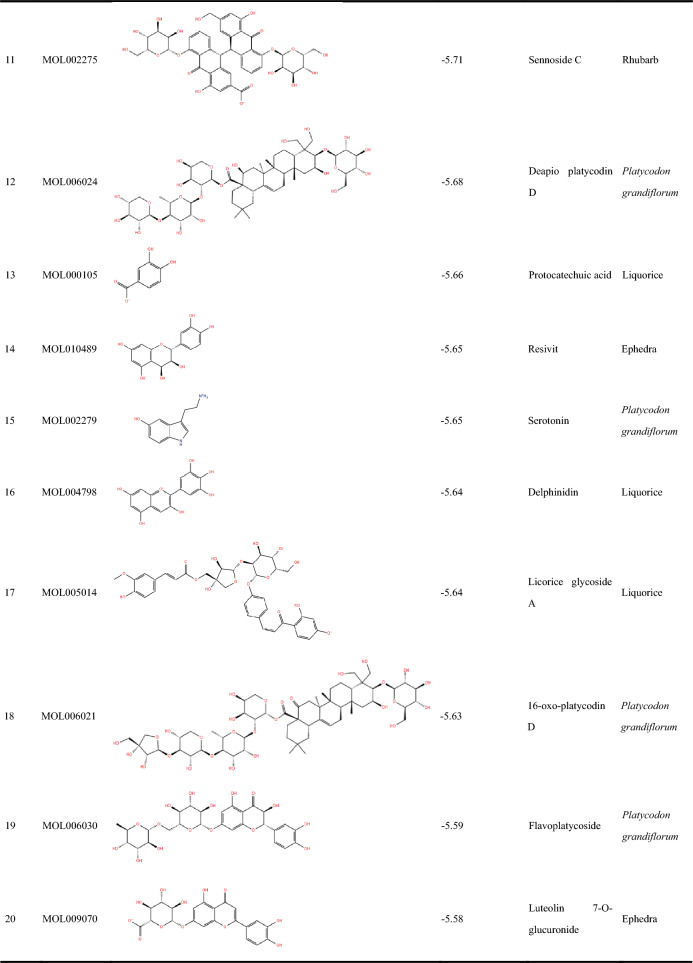

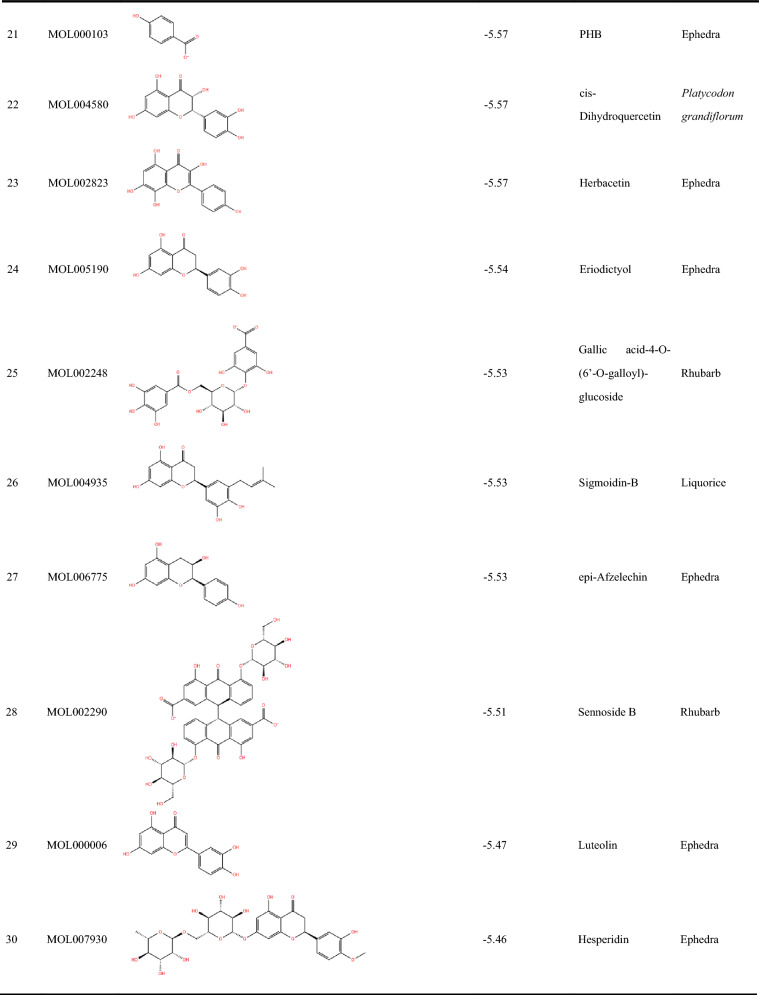
Compounds with top 30 docking scores

### Inhibitory activity of delphinidin and DPD against the SARS-CoV-2-S pseudovirus

**Fig. 3:**
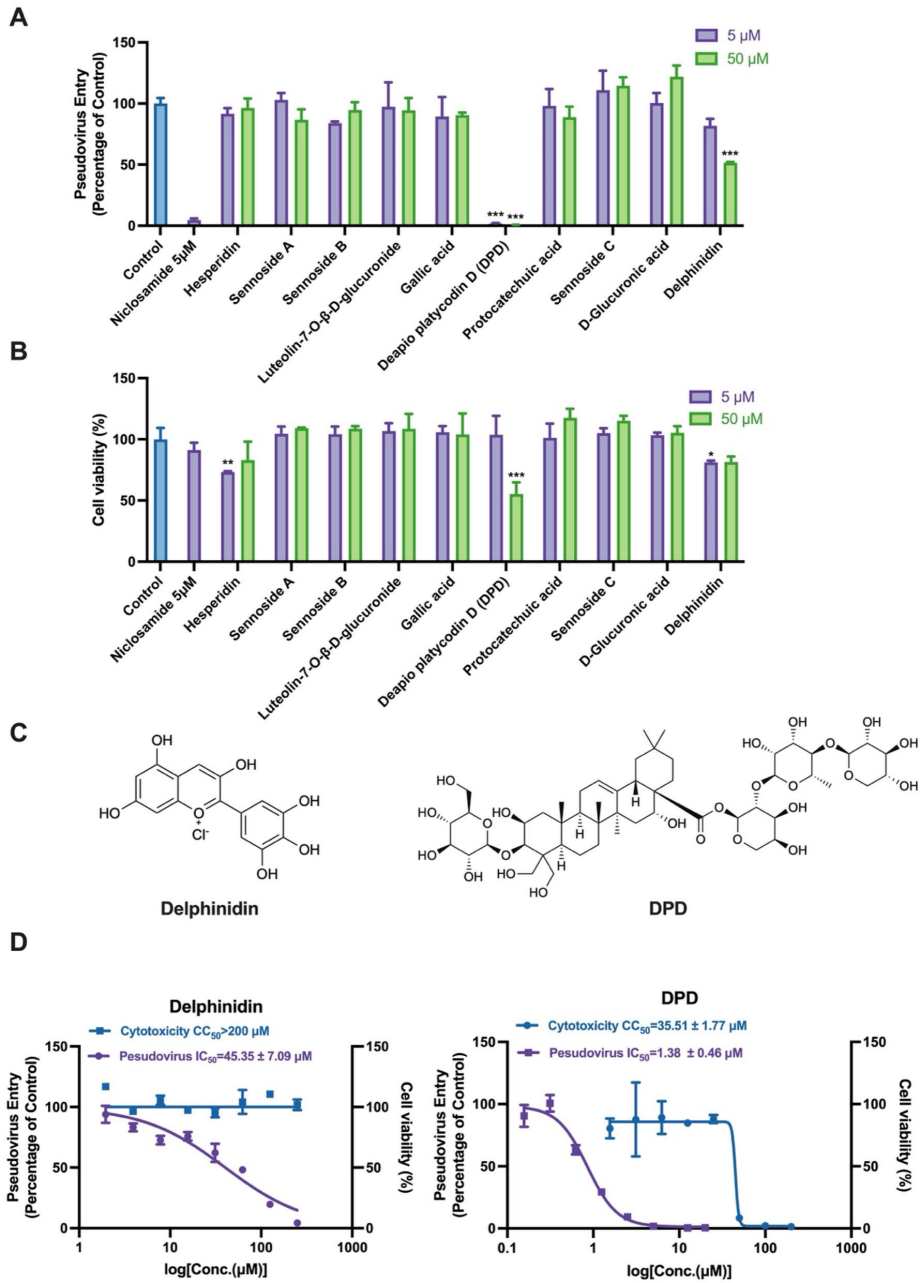
Compound screening and assessment of anti-SARS-CoV-2-S pseudovirus efficacy and cytotoxicity.** A**, **B** The antiviral activity and cytotoxicity of hesperidin, sennoside A, sennoside B, luteolin 7-O-glucuronide, gallic acid, deapio platycodin D (DPD), protocatechuic acid, sennoside C, D-glucuronic acid, delphinidin and the positive compound niclosamide. **C** Chemical structures of delphinidin and DPD. **D** IC_50_ and CC_50_ values of delphinidin and DPD. Results are shown as mean values with standard deviation (SD). Statistical significance was evaluated using P-values, with differences marked as significant at **p < 0.01 and highly significant at ***p < 0.001

We began our study by conducting a literature review of the top 30 compounds, excluding those that had already been tested for antiviral activity. Ultimately, we selected 10 commercially available compounds for experimental evaluation to assess their potential against SARS-CoV-2. The antiviral effects were evaluated using a SARS-CoV-2-S pseudovirus model at concentrations of 5 µM and 50 µM. As shown in Fig. 3A, delphinidin and DPD significantly inhibited the attachment of the SARS-CoV-2-S pseudovirus to ACE2-expressing HEK293T cells at these concentrations (*p* < 0.001). Additionally, cytotoxicity tests were conducted to exclude the possibility of false positives due to toxicity. As shown in Fig. 3B, delphinidin was not significantly cytotoxic, while DPD had cytotoxicity against hACE2-HEK293T cells at 50 µM (*p* < 0.001) and had antiviral activity but no cytotoxicity at 5 µM.

We subsequently conducted comprehensive assessments of the antiviral activity of these two compounds and their cytotoxic effects on hACE2-HEK293T cells. As depicted in Fig. 3D, both delphinidin and DPD (the structures displayed in Fig. 3C) inhibited SARS-CoV-2-S pseudovirus in a dose-dependent manner, with IC_50_ values of 45.35 ± 7.09 µM and 1.38 ± 0.46 µM, respectively. Crucially, the half-maximal cytotoxic concentration (CC_50_) for delphinidin exceeded 200 µM, while the CC_50_ value for DPD was 35.51 ± 1.77 µM. The selectivity index (SI), calculated as [CC_50_]/[IC_50_], was notably higher for DPD (SI = 25.73) compared to delphinidin (SI = 4.41), suggesting DPD as a more promising antiviral candidate.

### Delphinidin and DPD blocking the interaction of ACE2/S-RBD

**Fig. 4:**
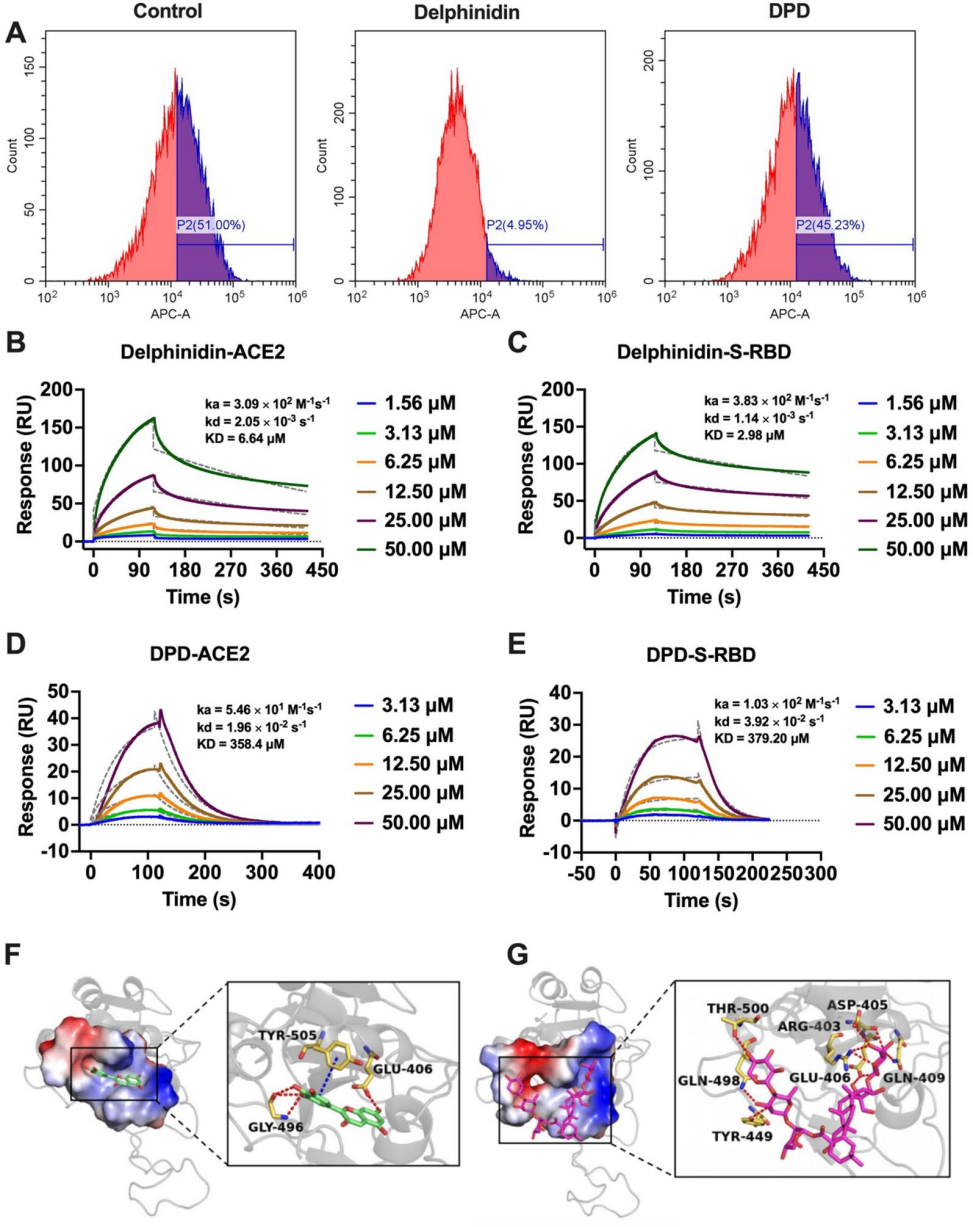
Compounds blocking ACE2/S-RBD interaction and the mechanism.** A** Flow cytometry determined the effecy of 100 µM delphinidin and 10 µM DPD disrupting the binding of soluble His-S-RBD to Vero-E6 cells. **B**–**E** SPR binding assays were performed to evaluate the binding kinetics and affinity of delphinidin (**B**, **C**) and DPD (**D**, **E**) with ACE2 and S-RBD. **F** Predicted binding mode of delphinidin with the S-RBD of SARS-CoV-2. **G** Predicted binding mode of DPD with the S-RBD of SARS-CoV-2 (PDB ID:6M0J). Carbon atoms of delphinidin are displayed in green, DPD is shown as magenta sticks, nitrogen atoms are in blue, and oxygen atoms in red. Key residues are shown as sticks. Red and blue dashes present hydrogen bonds and Pi-pi stacking interaction, respectively. Figures are generated with PyMOL [40]

Since the pseudovirus system is pseudotype SARS-CoV-2 strains infecting hACE2-293 T cells, we selected Vero-E6 cells endogenously expressing ACE2 for further exploring the mechanism of delphinidin and DPD. Using flow cytometry, we investigated the impact of these compounds on the binding of His-S-RBD to Vero-E6 cells. As shown in Fig. 4A, delphinidin and DPD both reduced the binding signal, with delphinidin showing particularly strong inhibition.

To identify whether delphinidin and DPD target S-RBD or ACE2, we used SPR to assess their binding affinity to both proteins. Delphinidin exhibited dose-dependent binding to ACE2, with a KD of 6.64 µM. Meanwhile, the binding rate constant (ka) of delphinidin with ACE2 was 3.09 × 10^2^ M^−1^ s^−1^, and the dissociation rate constant (kd) was 2.05 × 10^–3^ s^−1^, indicating slow binding and dissociation characteristics (Fig. 4B). The ka, kd and KD of delphinidin are 3.83 × 10^2^ M^−1^ s^−1^, 1.14 × 10^–3^ s^−1^, and 2.98 µM respectively, displaying slow binding and slow dissociation with S-RBD (Fig. 4C). In comparison, DPD showed lower binding affinity to both ACE2 and S-RBD (Fig. 4D, E).

As depicted in Fig. 4F, delphinidin is fully embedded within the binding pocket of SARS-CoV-2 S-RBD. The two phenolic hydroxyl groups of delphinidin form three hydrogen bonds with G496, while another phenolic hydroxyl group establishes a hydrogen bond with E406. Moreover, the phenyl ring forms pi-pi stacking with Y505. These residues in the binding pocket of SARS-CoV-2 S-RBD are identified as the key residues that interact with ACE2 [33, 39].

DPD interacts with the surface residues of SARS-CoV-2 S-RBD mainly through hydrogen bonds, including R403, D405, E406, Q409, Y449, Q498, and T500 (Fig. 4G). Among these residues, DPD forms two hydrogen bonds with R403, D405 and E406 respectively, and one hydrogen bond each with the remaining residues. In addition, hydrophobic interactions are formed between DPD and Y505. The interactions of DPD with these key residues may significantly contribute to its potent inhibitory potential against SARS-CoV-2 S protein to ruin its interaction with ACE2. This also explains why it interferes with the interaction between SARS-CoV-2 S-RBD and ACE2.

### Delphinidin and DPD inhibition of SARS-CoV-2-S variant pseudovirus entry into hACE2-HEK293T cells

**Fig. 5:**
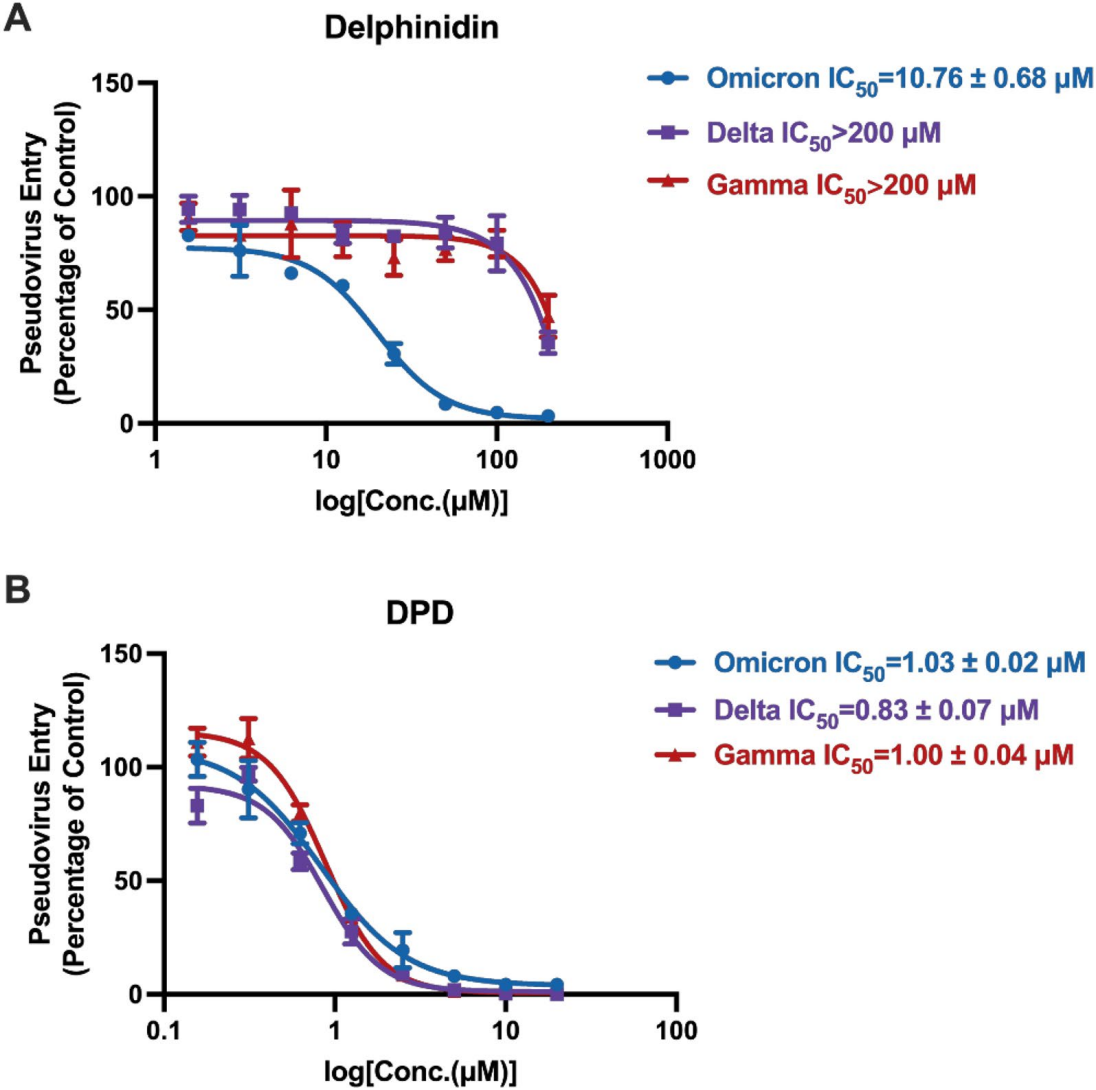
The antiviral effects of delphinidin and DPD against SARS-CoV-2-S pseudovirus variants, including Delta, Omicron, and Gamma.** A**, **B** Delphinidin (**A**) and DPD (**B**) effectively blocked the infection by various pseudotypes of SARS-CoV-2 variants in hACE2-HEK293T cells. The data are reported as mean ± standard deviation (SD)

We utilized a lentiviral system to generate pseudovirus versions of SARS-CoV-2 variants, including Delta, Gamma, and Omicron, to evaluate the antiviral effects of delphinidin and DPD. Delphinidin showed selective antiviral activity against the Omicron variant, with an IC_50_ value of 10.76 ± 0.68 µM, which was higher than that observed for the wild-type strain (Fig. 5A). DPD exhibited comparable antiviral activity against all tested pseudovirus strains, with IC_50_ values ranging from 0.83 ± 0.07 to 1.03 ± 0.02 µM (Fig. 5B). These findings highlight the potential of delphinidin and DPD as therapeutic agents for combating SARS-CoV-2 variants.

**Fig. 6:**
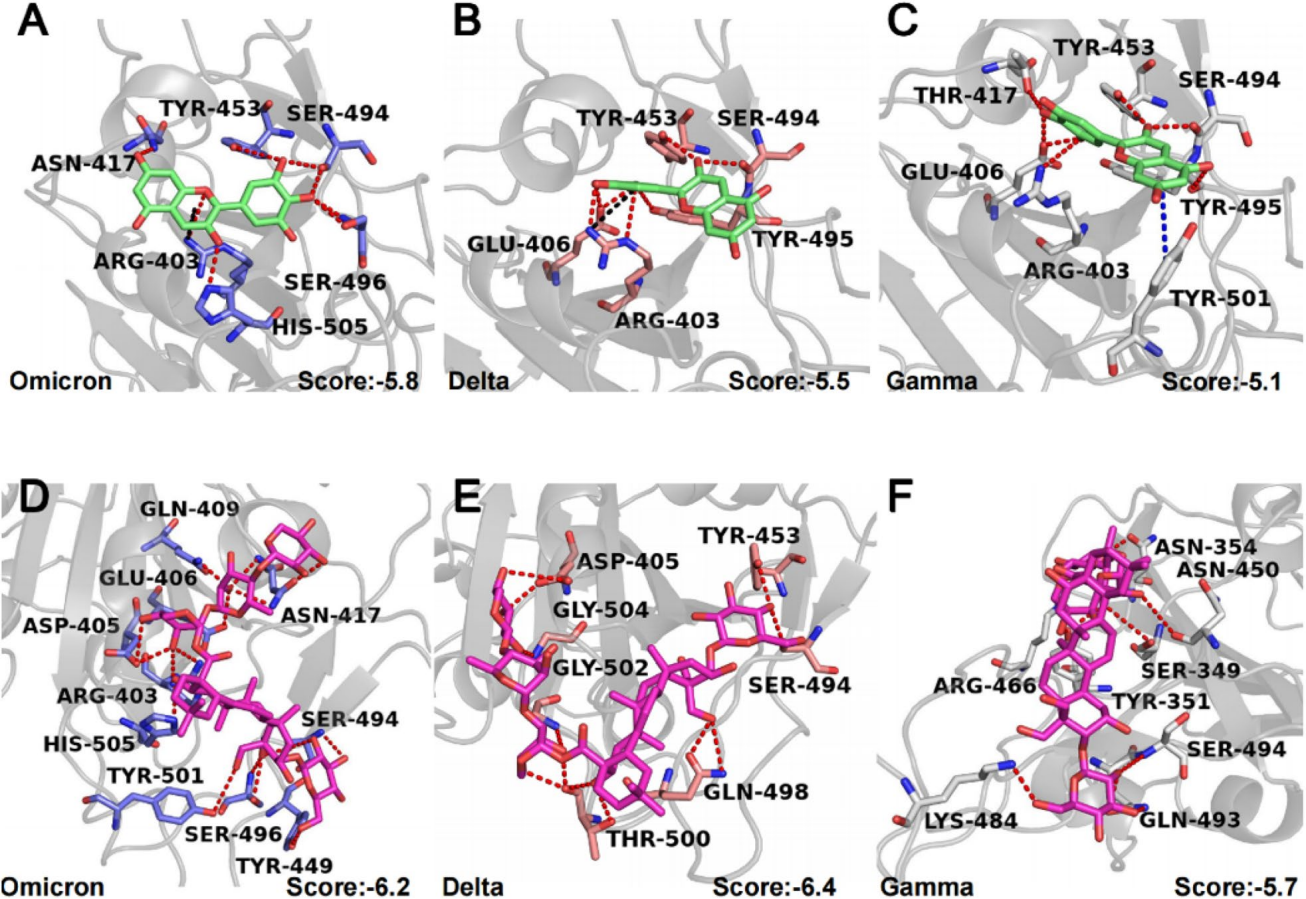
Predicted binding modes of delphinidin and DPD with three variants of SARS-CoV-2.** A**–**C** Key residues on (**A**) Omicron S-RBD (PDB:7T9L), (**B**) Delta S-RBD (PDB:7TEW), and (**C**) Gamma S-RBD (PDB:7NXC) are shown as sticks and labeled appropriately. Red, blue, and black dashes represent H bonds, pi-pi stacking interaction, and pi-cation, respectively. The corresponding average docking score is given at the bottom right of the graph. **D**–**F** Red dashes represent H bonds between variant S-RBD and DPD. The key residues are labeled in (**D**) Omicron S-RBD (PDB:7T9L), (**E**) Delta S-RBD (PDB:7TEW), and (**F**) Gamma S-RBD (PDB:7NXC)

To figure out the binding mode of small molecules and mutant strains, we adopted the same docking procedure on the other three mutants. The results show that delphinidin and Omicron exhibited the best docking score, followed by the Delta variant, with the Gamma variant ranking last. For the Omicron variant, delphinidin interacts with several key residues, including R403 and H505 (Fig. 6A). R403 serves as a pivotal role in SARS-CoV-2 S-RBD’s recognition of the host ACE2 through a salt bridge with E37, and R403 forms an additional pi-cation interaction with delphinidin in both Omicron and Delta variants (Fig. 6A, B). Meanwhile, SARS-CoV-2 S-RBD’s Y505 is hydrogen-bonded to ACE2’s E37, and it interacts with the hydrophobic part of ACE2’s K353 [41]. Interestingly, H505 is also predicted to be one of the key residues interacting with delphinidin in Omicron. However, no similar interaction exists in Delta and Gamma variants. Besides, both K417N and K417T mutations in SARS-CoV-2 S-RBD decrease the binding affinity with hACE2 [42]. Our predicted binding modes showed that delphinidin has interactions with N417 and T417 in Omicron and Gamma variants (Fig. 6A, C), respectively, whereas not in Delta. We suppose that simultaneous interactions involving H505, N417 and R403 are the key to the inhibition effect of delphinidin. As a result, the ligand exhibits the strongest inhibitory efficacy against Omicron, which is consistent with our experimental results (Fig. 6A).

As shown in Fig. 6D–F, DPD mainly interacts with three mutants through hydrogen bonds, forming a total of seventeen hydrogen bonds with Omicron, thirteen with Delta, and ten with Gamma. In the Delta variant, DPD has hydrophobic interactions with Y505 (Fig. 6D). Although DPD forms the fewest hydrogen bonds with S-RBD from SARS-CoV-2 Gamma variant, it can form hydrophobic interactions with F490 in the Gamma mutant. Additionally, DPD forms a salt bridge with K484 (Fig. 6F). Thus, DPD is able to inhibit all three SARS-CoV-2 variants.

### Delphinidin—an allosteric inhibitor of SARS-CoV-2 3CL^pro^

**Fig. 7:**
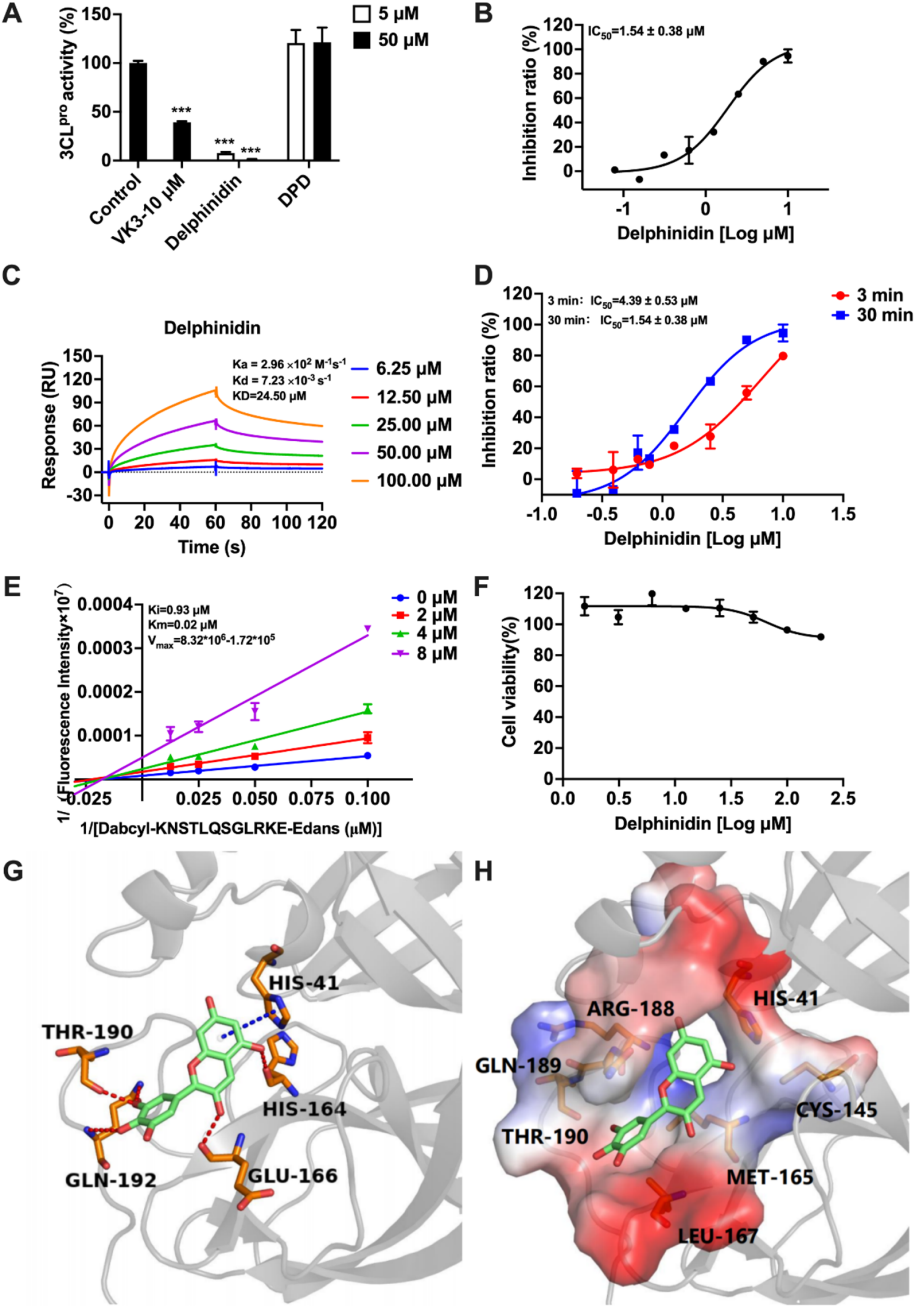
Inhibitory effect of delphinidin on SARS-CoV-2 3CL^pro^ and inhibitory mechanism.** A** Inhibition of SARS-CoV-2 3CL^pro^ by delphinidin and DPD. **B** The IC_50_ value for delphinidin's inhibition of SARS-CoV-2 3CL^pro^. **C** The binding interaction between delphinidin and SARS-CoV-2 3CL^pro^ measured by SPR. **D** TDI of SARS-CoV-2 3CL^pro^ by delphinidin. **E** Lineweaver–Burk plots for delphinidin’s inhibitory mechanism. **F** Cytotoxicity of delphinidin to Vero-E6 cells. **G** Key interactions involved in stabilizing delphinidin at the binding site. Delphinidin is depicted as green sticks, and key residues are colored in orange. Hydrogen bonds are shown as red lines, and the pi−pi stacking interaction involving residue His41 is also highlighted in blue. **H** Interactions of delphinidin with SARS-CoV-2 3CL^pro^ displayed in vacuum electrostatics. The data are expressed as the mean ± standard deviation (SD). Statistical significance was evaluated using P-values, with differences considered highly significant at ***p < 0.001

To confirm the target selectivity of delphinidin and DPD, we employed a FRET-based assay against SARS-CoV-2 3CL^pro^. Vitamin K3 (VK3) was used as a positive control based on a previously reported method [35]. Interestingly, only delphinidin demonstrated significant inhibition of SARS-CoV-2 3CL^pro^ (Fig. 7A), with an IC_50_ of 1.54 ± 0.38 µM (Fig. 7B). We further confirmed delphinidin’s binding to SARS-CoV-2 3CL^pro^ via SPR, revealing Ka = 2.96 × 10^2^ M^−1^ s^−1^, Kd = 7.23 × 10^–3^ s^−1^ and the KD value of 24.50 µM (Fig. 7C). Time-dependent inhibition (TDI) assays were then conducted to investigate the inhibition mechanism [35]. The IC_50_ shifted significantly (by 2.85 times, from 4.39 to 1.54 µM) after pre-incubation with 3CL^pro^ for 3 or 30 min, suggesting that delphinidin does not covalently bind to the protease (Fig. 7D). Then, the specific binding mechanism of delphinidin to SARS-CoV-2 3CL^pro^ was explored by Lineweaver–Burk double reciprocal plots (Fig. 7E). V_max_ value was 8.32 × 10^6^–1.72 × 10^5^ RFU/min varied with the concentration of delphinidin, showing that delphinidin acts as a non-competitive inhibitor, with a Ki of 0.93 µM and Km value of 0.02 μM. Cytotoxicity testing on Vero-E6 cells, commonly used in SARS-CoV-2 studies, showed no toxicity even at 200 µM, indicating that delphinidin is a potentially safe therapeutic agent (Fig. 7F).

To investigate the molecular mechanism of action of delphinidin in inhibiting SARS-CoV-2 3CL^pro^, we performed molecular docking of the compound and the protease. As illustrated in Fig. 7G, delphinidin interacts with the catalytic site residues of SARS-CoV-2 3CL^pro^ through hydrogen bonds, pi-stacking, and hydrophobic interactions. The two hydroxyl groups of benzopyrylium form hydrogen bonds with the mainchain of H164 and E166, respectively, which nips M165 and forms hydrophobic interaction with it. Meanwhile, delphinidin forms a pi–pi interaction with H41 of the catalytic Cys145-His41 dyad. In Fig. 7H, the benzopyrylium ring is inserted into the active site by hydrophobic interactions with R188, Q189, and H41, and the benzene ring fills the gap between T190 and L167. Although direct contact between delphinidin and C145 is absent, its interaction with surrounding residues could partly obstruct substrate access to the catalytic dyad within the active site. Consequently, delphinidin is firmly bound in the catalytic site of SARS-CoV-2 3CL^pro^ while interacting with the catalytic residue E166, H41 and R190, Q192 at the active site, which are the key elements for the recognition of substrates and peptidomimetic inhibitors [43]. Thus, consistent with the conclusion of experiments, delphinidin is a noncompetitive inhibitor.

Similar to delphinidin, we also applied the same docking procedure on SARS-CoV-2 3CL^pro^ and DPD. It was observed that DPD failed to be docked in the catalytic pockets, possibly due to the large size of DPD, which might explain its lack of inhibition against SARS-CoV-2 3CL^pro^.

### Selectivity of delphinidin

**Fig. 8:**
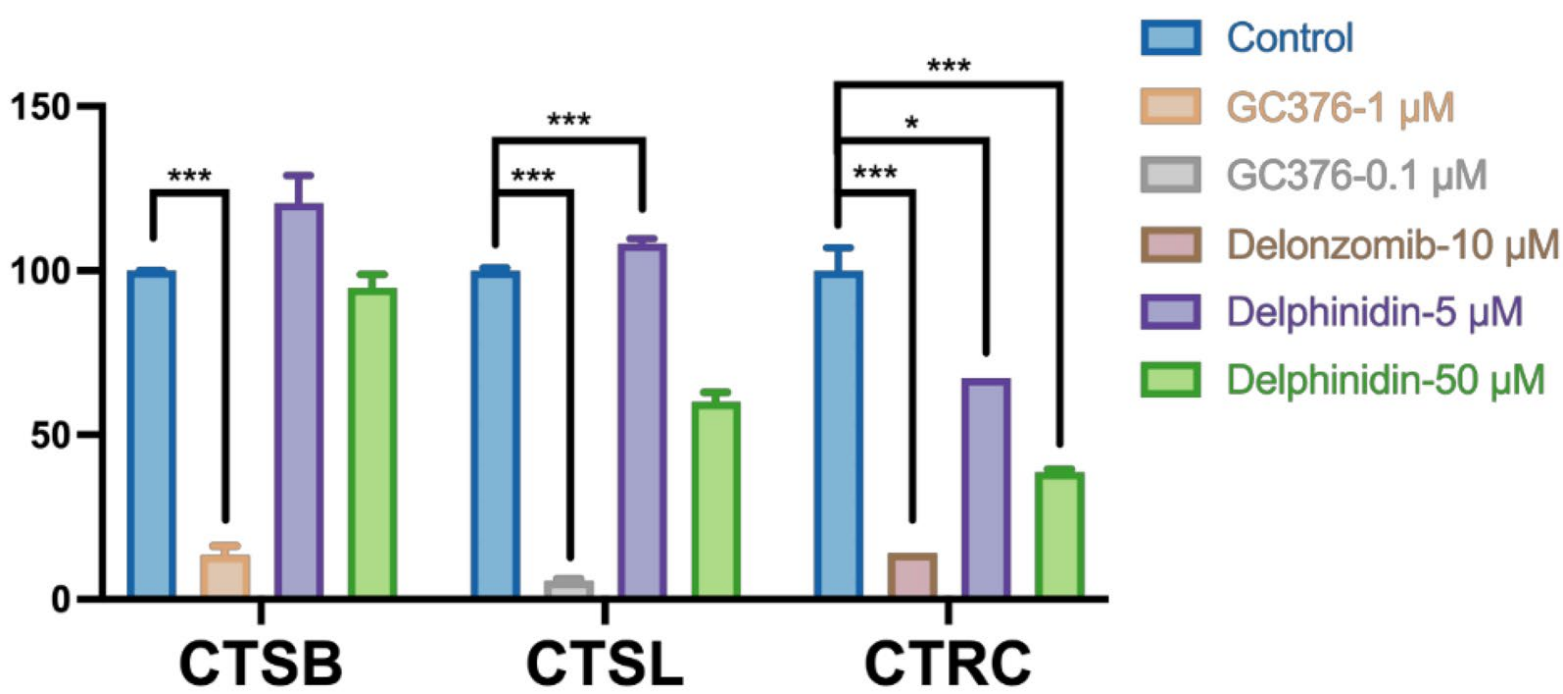
Selectivity of delphinidin. Inhibition of delphinidin to CTSB, CTSL, CTRC. The data are reported as mean ± standard deviation (SD). Statistical significance was evaluated using P-values, with * *p* < 0.05 and *** *p* < 0.001 indicating different levels of significance

Covalent inhibitors targeting SARS-CoV-2 3CL^pro^ can potentially cause off-target effects by binding to and inhibiting cathepsins, which may lead to unwanted side effects [44]. To evaluate delphinidin’s selectivity, we tested its impact on three host proteases: CTSB, CTRC, and CTSL. Earlier studies have reported that GC376 can inhibit both CTSB and CTSL [44]. Delanzomib served as a positive control for CTRC inhibition [45]. As illustrated in Fig. 8, delphinidin did not inhibit CTSB but showed a strong inhibitory effect on CTSL and CTRC (*p* < 0.001) at a concentration of 50 µM. These results suggest that additional structure–activity relationship research on delphinidin is necessary to improve its specificity.

### DPD blocking HCoV-229E in vitro

Next, it is necessary to assess the antiviral effects of delphinidin and DPD in SARS-CoV-2-infected cells within a P3 laboratory setting. However, our current facilities do not meet the necessary conditions to conduct this specific viral experiment. Since detection using a live virus is more convincing, we have selected HCoV-229E, which causes mild upper respiratory tract disease, to evaluate the antiviral activities of our candidates against coronaviruses. Despite the limited overall sequence homology between SARS-CoV-2 and HCoV-229E 3CL^pro^, the active sites of these enzymes exhibit a high degree of conservation [37]. Moreover, several 3CL^pro^ inhibitors have demonstrated consistent antiviral efficacy against both SARS-CoV-2 and HCoV-229E [46]. Therefore, HCoV-229E serves as a suitable surrogate for SARS-CoV-2 in live virus experiments.

**Fig. 9:**
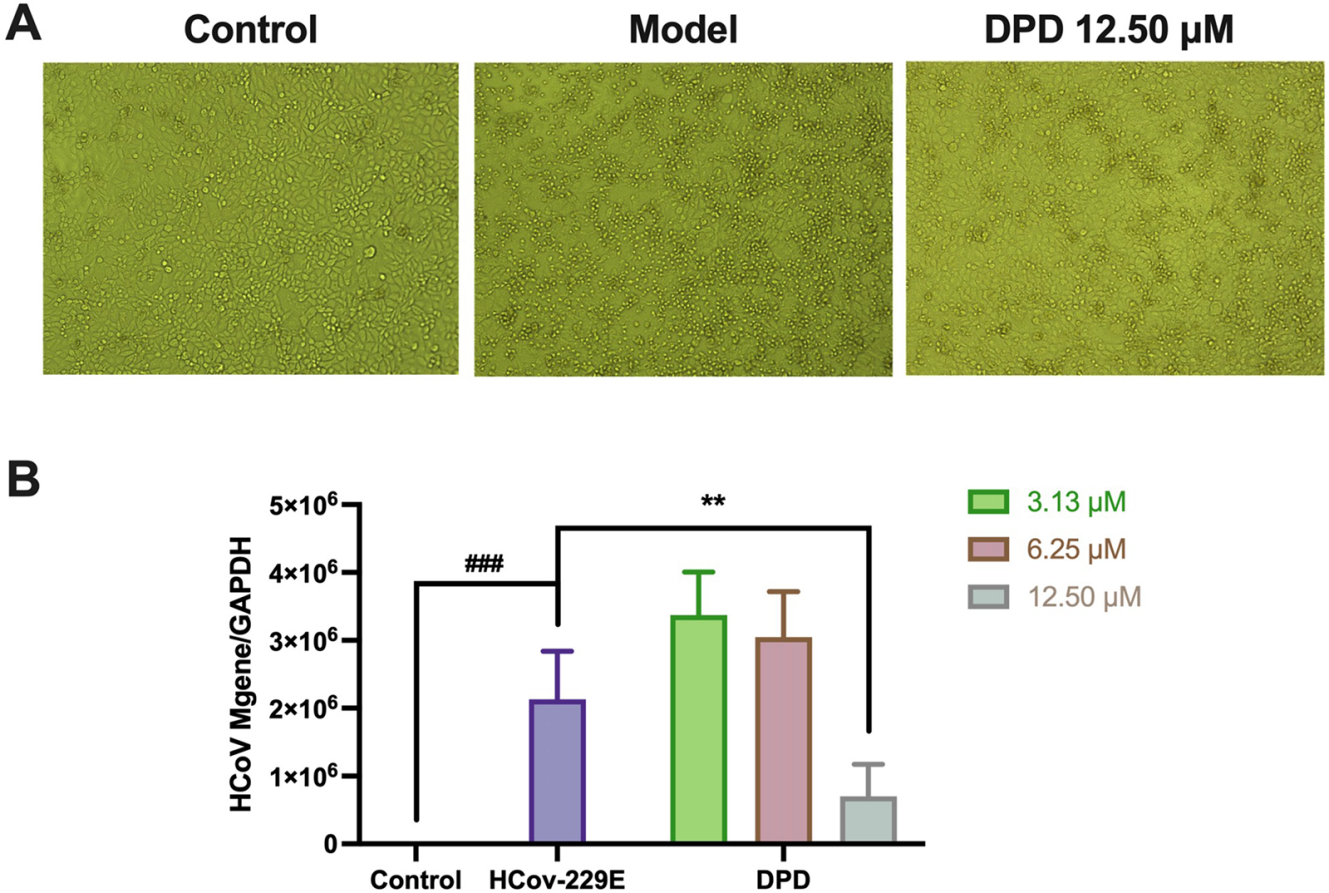
The anti HCoV-229E coronavirus activity of DPD.** A** The diagram displayed the CPE in BEL-7402 cells, including those of normal cells, the virus-only treated model, and the 12.50 µM DPD treated groups. **B** The inhibition of DPD on high HCoV-229E viral load replication in BEL-7402 cells was measured by RT-qPCR. Data are expressed as mean ± standard deviation (SD). Statistical significance was assessed using P-values, with thresholds set at **p < 0.01 and ^###^*p* < 0.001

As depicted in Fig. 9A, BEL-7402 cells infected with the HCoV-229E virus exhibited CPE, leading to cell death. The MNTC of DPD for BEL-7402 cells was determined to be 12.50 µM through continuous monitoring of CPE following treatment with a range of DPD concentrations. Moreover, DPD demonstrated significant antiviral activity against HCoV-229E at this MNTC concentration of 12.50 µM (Fig. 9B). This finding indicates that DPD can effectively inhibit viral infection, suggesting its potential as a therapeutic agent against HCoV-229E infection. As previously noted, delphinidin was identified as a dual inhibitor targeting both the SARS-CoV-2-S pseudovirus and 3CL^pro^, while DPD displayed antiviral effects against HCoV-229E in vitro, highlighting their potential as COVID-19 therapeutic candidates.

## Discussion

Ephedra and liquorice, *Platycodon grandiflorum*, and rhubarb derived from QFPDD have been widely utilized for treating infectious diseases characterized by respiratory and digestive symptoms. Till now, there remains a paucity of evidence on QFPDD’s effectiveness in managing these symptoms in COVID-19 patients and its active ingredients against SARS-CoV-2 have yet to be discovered.

In this study, we efficiently screened the ingredients from the clinically effective herb by combining the TCM theory and experiments. We aimed to elucidate the mechanism of action for individual herbs and identified delphinidin and DPD as promising candidates against SARS-CoV-2 through pseudovirus neutralization assays (Fig. 3A, B). Delphinidin stands out as one of the most valuable polyphenolic anthocyanins, well-regarded for its significant biological activities. It is a widely occurring anthocyanin pigment present in vibrant vegetables and fruits like grapes, berries and pigmented cabbage, as well as dietary supplements [47]. Recently, numerous studies have underscored the various health-promoting attributes of delphinidin, including antioxidant, anti-inflammatory, neuroprotective, anti-adipogenic, cardiovascular protective, and anti-cancer effects [48–54]. Delphinidin has also demonstrated potential in combatting Flaviviruses like the dengue virus, West Nile virus, hepatitis C virus and Zika virus [47, 55–57]. Delphinidin displayed neutralizing activity against the Omicron strain (IC_50_ = 10.76 ± 0.68 µM) (Fig. 5A). These experimental findings align with our calculated binding pattern analysis. For the Omicron variant, delphinidin interacts with several key residues, including R403 and H505 (Fig. 6A). R403 is crucial for S-RBD to recognize the ACE2 receptor, forming a salt bridge with E37, and in Omicron and Delta variants, this interaction is strengthened by an additional pi-cation interaction with delphinidin (Fig. 6A, B). Meanwhile, Y505 in S-RBD forms a hydrogen bond with ACE2’s E37 and interacts with the hydrophobic part of ACE2’s K353 [41]. Interestingly, H505 is also predicted to be one of the key residues interacting with delphinidin in Omicron. However, no similar interaction exists in Delta and Gamma mutants. Our previous research revealed that R403 is vital for S-RBD recognition of ACE2 by forming a salt bridge with ACE2’s E37 [29]. Meanwhile, SPR experiments showed that a single Y505A mutation abolishes ACE2 binding, emphasizing the critical role of Y505 in specificity [29]. Since 3CL^pro^ is critical to the SARS-CoV-2 life cycle, it serves as a key therapeutic target. To verify the target selectivity of delphinidin, we employed our well-established 3CL^pro^ activity screening platform to evaluate its anti-3CL^pro^ activity. Moreover, Vero-E6 cells, commonly used in in vitro SARS-CoV-2 infection studies, showed that delphinidin has no notable cytotoxicity (CC_50_ > 200 µM)  (Fig. 5A), supporting its potential as an antiviral agent against SARS-CoV-2.

In contrast, the pharmacological properties of DPD from *Platycodon grandiflorum* have been relatively underexplored. This study is the first to demonstrate DPD’s antiviral activity against pseudotyped SARS-CoV-2 and its variants with different spike protein mutations, achieved by inhibiting the interaction between ACE2 and S-RBD. DPD showed significantly stronger neutralizing effects against three mutant strains, with IC_50_ values ranging from 0.83 ± 0.07 to 1.03 ± 0.02 µM (Fig. 5B). Mechanistic investigations revealed that DPD blocks the binding of S-RBD to ACE2 through hydrogen bonding and hydrophobic interactions with key residues. Molecular docking further clarified DPD’s binding mode with SARS-CoV-2 variants, shedding light on the interactions responsible for its inhibitory action. Notably, DPD also effectively inhibited the viral activity of HCoV-229E, a virus with a high degree of homology to SARS-CoV-2, underscoring its potential as a therapeutic candidate for COVID-19. DPD presents a promising lead for the development of small-molecule drugs against COVID-19. However, further optimization is necessary to improve its potency and extend its antiviral efficacy to other circulating coronavirus variants.

Delphinidin’s dual inhibition of 3CL^pro^ and the S-RBD/ACE2 interaction aligns with recent findings on structurally similar catechol/pyrogallol derivatives, such as delphinidin-3-glucoside, which covalently binds 3CL^pro^ to suppress viral replication [58]. Notably, our computational analysis revealed that delphinidin preferentially interacts with Omicron-specific residues (e.g., R403 and H505), potentially explaining its strain-dependent efficacy. This dual-target mechanism resonates with emerging strategies to simultaneously block viral entry and replication, enhancing resilience against mutational evasion.

Interestingly, while DPD (structurally analogous to platycodin D) shares a triterpenoid backbone with platycodin D, its antiviral mechanism diverges significantly. Unlike platycodin D, which inhibits lysosome/TMPRSS2-dependent viral entry by redistributing membrane cholesterol [24], DPD directly disrupts S-RBD/ACE2 binding through hydrogen bonding and hydrophobic interactions with key spike residues. This distinction suggests that subtle structural modifications, such as glycosylation patterns or side-chain substitutions, may redirect natural analogs toward distinct molecular targets—either host membrane components (platycodin D) or viral surface proteins (DPD). Such divergence underscores the importance of structural specificity in natural product-driven antiviral design.

Delphinidin, a flavonoid anthocyanidin, has garnered attention due to its potential therapeutic benefits, including antioxidant and anti-inflammatory properties [54]. Research indicates that delphinidin undergoes rapid metabolism in the gastrointestinal tract and liver [54]. This process involves the activity of catechol-O-methyltransferase (COMT), which methylates the 4′-OH group on the B-ring, leading to metabolites with different distribution profiles. Despite these metabolic transformations, delphinidin’s bioavailability remains low [54]. In clinical studies, Delphinol® (a maqui berry extract rich in delphinidin glycosides) have shown that delphinidin glycosides (e.g., Dp-3G) exhibit slightly improved bioavailability, but delphinidin itself still faces challenges in terms of absorption and systemic concentration [54, 59].

In contrast, DPD exhibits distinct pharmacokinetic behavior that may offer better therapeutic potential. This triterpenoid saponin from *Platycodon grandiflorum* is characterized by a rapid absorption rate, with Tmax values consistently under 1 h, indicating rapid uptake following oral administration [60]. Furthermore, it is noteworthy that DPD demonstrates slow elimination from tissues, particularly lung tissue, indicating prolonged activity [60]. This behavior could be particularly advantageous for treating respiratory diseases like COVID-19, where sustained lung tissue concentration is vital for efficacy. The pharmacokinetic studies involving DPD also highlight the impact of glycoside side chains on absorption and distribution [61]. The variations in glycoside chain length and position influence the compound’s tissue distribution, underscoring the importance of structural modifications for enhancing pharmacokinetics. However, similar to delphinidin, DPD’s bioavailability could still be impacted by factors such as gut microbiota interactions and enzymatic activity [61]. Research suggests that the presence of specific bacterial populations, such as Lactobacillus, may facilitate the hydrolysis of glycosidic bonds, potentially enhancing the absorption of DPD.

In conclusion, both delphinidin and DPD present interesting therapeutic potential, but their low oral bioavailability limits their effectiveness. The ongoing exploration of formulation strategies (e.g., nanoformulations, liposomal delivery) and the role of gut microbiota in modulating their pharmacokinetics may offer pathways to improving their therapeutic applicability in treating infectious diseases like COVID-19.

At present, dual-target antiviral strategies are gaining attention. A previous study demonstrated that compounds 7 and 13 inhibit both viral proteases, such as 3CL^pro^ and PL^pro^, as well as host proteases, employing a dual-inhibition strategy aimed at blocking viral replication and entry into host cells [62]. Another study explored the repurposing of FDA-approved antiviral drugs, identifying ganciclovir and zanamivir as dual inhibitors of the SARS-CoV-2 S protein and 3CL^pro^ through computational screening and molecular docking [63]. Similarly, compounds 6c and 7e have been identified as dual inhibitors of PL^pro^ and 3CL^pro^, making them promising leads for further antiviral drug development [64]. These discoveries are critical in the ongoing battle against COVID-19, especially in light of the virus’s mutations and the emergence of new variants. The dual-target approach offers a more comprehensive and long-lasting antiviral strategy. In conclusion, the identification of dual-target antiviral compounds in this study provides a novel class of broad-spectrum antiviral agents for COVID-19 prevention and treatment.

## Conclusion

To conclude, ephedra and liquorice in QFPDD, which historically use in infectious diseases, along with two other Chinese herbs—*Platycodon grandiflorum* and rhubarb, have aroused our interest. Both of these latter herbs demonstrated pseudovirus inhibitory activity. Initially, we identified delphinidin from ephedra and DPD from *Platycodon grandiflorum* as promising candidates for combating SARS-CoV-2 through pseudovirus neutralization assays. Delphinidin exhibited notable neutralizing activity against the Omicron strain but had no significant inhibitory effect on the Delta and Gamma mutants. Our findings align with molecular docking analyses, which revealed delphinidin’s interaction with crucial residues such as R403 and H505 specific to the Omicron variant, whereas such interactions were absent in the Delta and Gamma mutants. In addition, delphinidin exhibited the capacity to inhibit 3CL^pro^ activity, reinforcing its potential as an antiviral therapeutic compound.​ Thus, the discovery of dual-target antiviral drugs in this study presents a novel research avenue for COVID-19 prevention and control strategies. Additionally, DPD exhibited superior neutralizing activity against three mutants than wildtype pesudovirus. We found that DPD formed hydrogen bonds and hydrophobic interactions with key residues, thereby interfering with the binding of S-RBD to ACE2. Additionally, DPD significantly inhibited the viral activity of HCoV-229E, indicating its potential as a treatment for COVID-19. Further optimization of DPD could improve its potency and antiviral efficacy, leading to stronger chemical compounds against diverse circulating coronavirus variants.

## Supplementary Information

Supplementary Material 1.

## Data Availability

The raw data supporting the conclusions of this article will be made available by the authors on request.
