# Supplementary material for: Exploring anti-SARS-CoV-2 natural products: dual-viral target inhibition by delphinidin and the anti-coronaviral efficacy of deapio platycodin D
Source: Nat Prod Bioprospect. 2025 Jun 13;15(1):39. doi: 10.1007/s13659-025-00523-w (PMC12165925; doi:10.1007/s13659-025-00523-w)
Supplement: Supplementary file 1 — Supplementary Material 1. [file 13659_2025_523_MOESM1_ESM.pdf]

Exploring anti-SARS-CoV-2 natural products: dual-viral target inhibition by delphinidin and the anti-coronaviral efficacy of deapio platycodin D

Jiani Lu[1,2,#], Yan Tang[3,#], Hongtao Li[1,2,#], Saisai Tian[1,4], Xixiang Chen[1], Xueyue Song[6], Pengcheng Qin[1,2,5], Jiangrong Xu[1], Haiyan Zhu[6], Liqiang Ni[7], Huarong Du[7,*], Weidong Zhang[1,4,*], Weihua Li[3,*], Lili Chen[1,2,*]

[1]The Research Center for Traditional Chinese Medicine, Shanghai Institute of infectious Diseases and Biosecurity, Shanghai Frontiers Science Center of TCM Chemical Biology, Institute of lnterdisciplinary Integrative Medicine Research, Shanghai University of Traditional Chinese Medicine. Shanghai, 201203, China

[2]Longhua Hospital, Shanghai University of Traditional Chinese Medicine, Shanghai, 201203, China

[3]Shanghai Frontiers Science Center of Optogenetic Techniques for Cell Metabolism, Shanghai Key Laboratory of New Drug Design, School of Pharmacy, East China University of Science and Technology, Shanghai, 200237, China

[4] School of Pharmacy, Second Military Medical University, Shanghai, 200433, China

[5]School of Pharmacy, Henan University, Kaifeng, 475001, China

[6]Department of Biological Medicines & Shanghai Engineering Research Center of ImmunoTherapeutics, School of Pharmacy, Fudan University

[7]Shanghai University of Traditional Chinese Medicine, Shanghai, 201203, China

*Correspondence:

Huarong Du

Shanghai University of Traditional Chinese Medicine, Shanghai, 201203, China

E-mail: huarongdu2017@shutcm.edu.cn

Weidong Zhang, Ph D

School of Pharmacy, Second Military Medical University, Shanghai, 200433, China; School of Pharmacy, Henan University, Kaifeng, 475001, China; Academy of Interdisciplinary Sciences, Shanghai University of Traditional Chinese Medicine, Shanghai, 201203, China
E-mail: wdzhangy@hotmail.com

Weihua Li, Ph D
Shanghai Frontiers Science Center of Optogenetic Techniques for Cell Metabolism, Shanghai Key Laboratory of New Drug Design, School of Pharmacy, East China University of Science and Technology, Shanghai, 200237, China
E-mail: whli@ecust.edu.cn

Lili Chen, Ph D
Shanghai Frontiers Science Center of TCM Chemical Biology, Institute of Interdisciplinary Integrative Medicine Research; Longhua Hospital, Shanghai University of Traditional Chinese Medicine, Shanghai, 201203, China
E-mail: llchen@shutcm.edu.cn

[#] The contribution of these authors to this work is equal.

Supplementary Table 1 The constituent herbs of QFPDD

Chinese Name	English Name	Latin Name
Bai Zhu	Atractylodes Macrocephala	*Atractylodis Macrocephalae Rhizoma*
Chai Hu	Bupleurum Root	*Bupleuri Radix*
Chen Pi	Aged Tangerine Peel	*Citri Reticulatae Pericarpium*
Fu Ling	Poria Cocos	*Poria*
Gui Zhi	Cinnamon Twig	*Cinnamomi Ramulus*
Huang Qin	Baikal Skullcap Root	*Scutellariae Radix*
Huo Xiang	Patchouli	*Agastache rugosus*
Jiang Ban Xia	Pinellia Rhizome (prepared with ginger)	*Pinelliae Rhizoma*
Kuan Dong Hua	Coltsfoot Flower	*Farfarae Flos*
Ma Huang	Ephedra	*Ephedrae Herba*
Shan Yao	Chinese Yam	*Dioscoreae Rhizoma*
She Gan	Belamcanda Rhizome	*Belamcandae Rhizoma*
Sheng Jiang	Fresh Ginger	*Zingiberis Rhizoma Recens*
Sheng Shi Gao	Raw Gypsum	*Raw Gypsum*
Xing Ren	Apricot Kernel	*Armeniacae Semen Amarum*
Xi Xin	Asarum	*Asari Radix et Rhizoma*
Ze Xie	Alisma Rhizome	*Alismatis Rhizoma*
Gan Cao	Liquorice	*Glycyrrhizae Radix et Rhizoma*
Zhi Shi	Immature Bitter Orange	*Fructus Aurantii Immaturus*
Zhu Ling	Polyporus	*Polyporus*
Zi Wan	Purple Aster Root	*Asteris Radix et Rhizoma*

Supplementary Figure 1. HPLC for deapio platycodin D

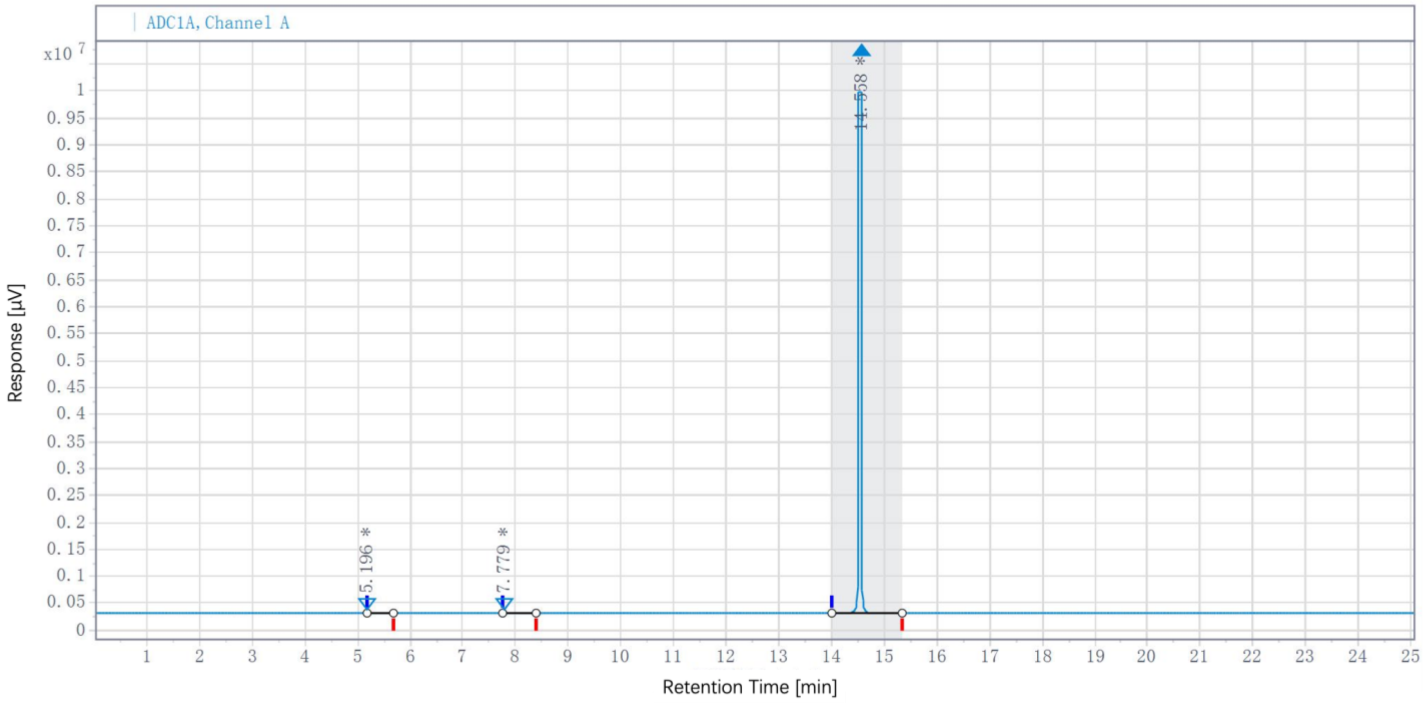

#	Retention Time (min)	Peak Area	Peak Area %	Peak Height
1	5.196	591.716	0.001	121.9
2	7.779	510.882	0.001	137.827
3	14.558	40300713.95	99.997	10799781.3

Supplementary Figure 2. UPLC-MS for delphinidin

Ret. Time: 0.66 <<<< POSITIVE SPECTRA >>>>

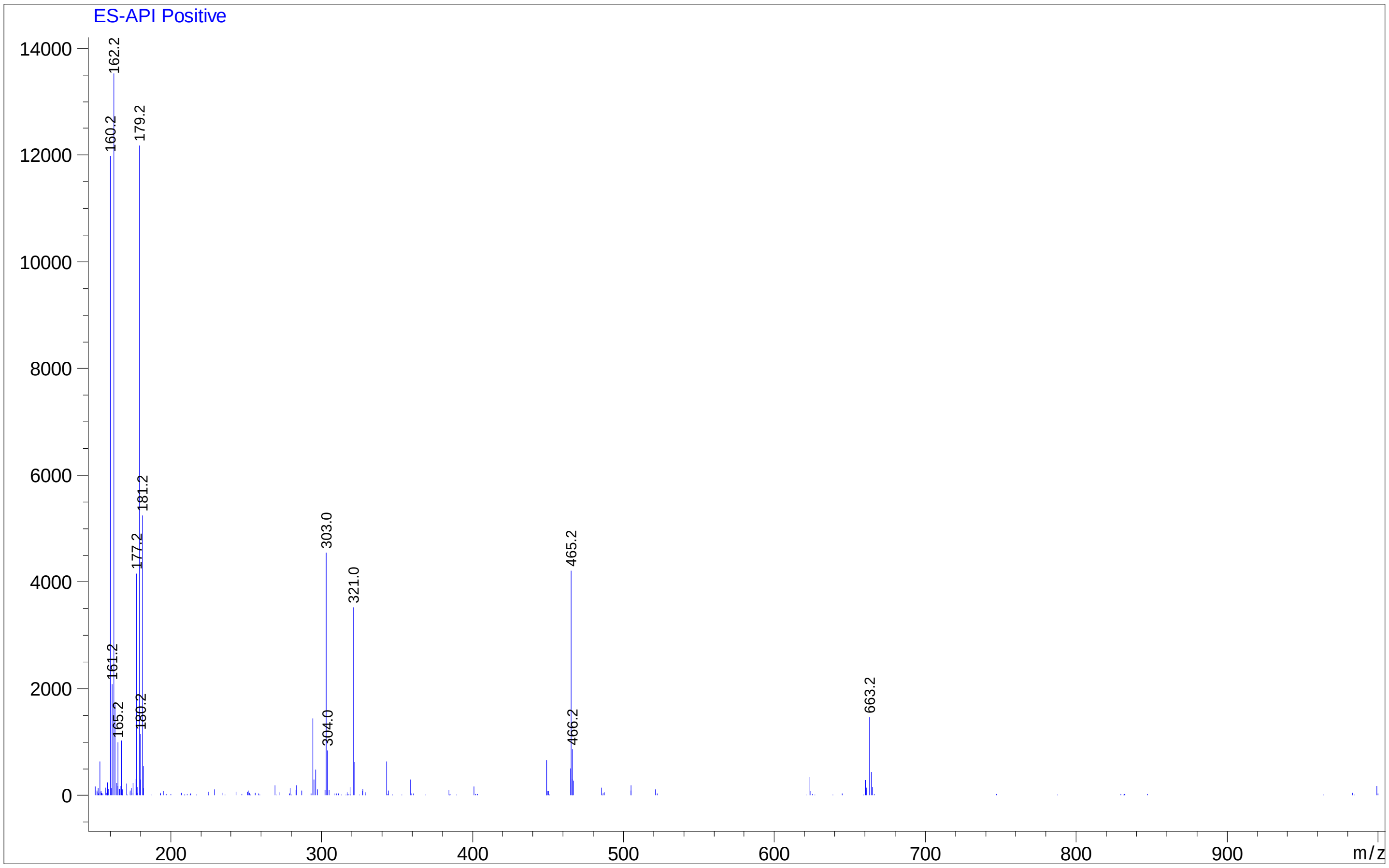

Ret. Time: 0.70

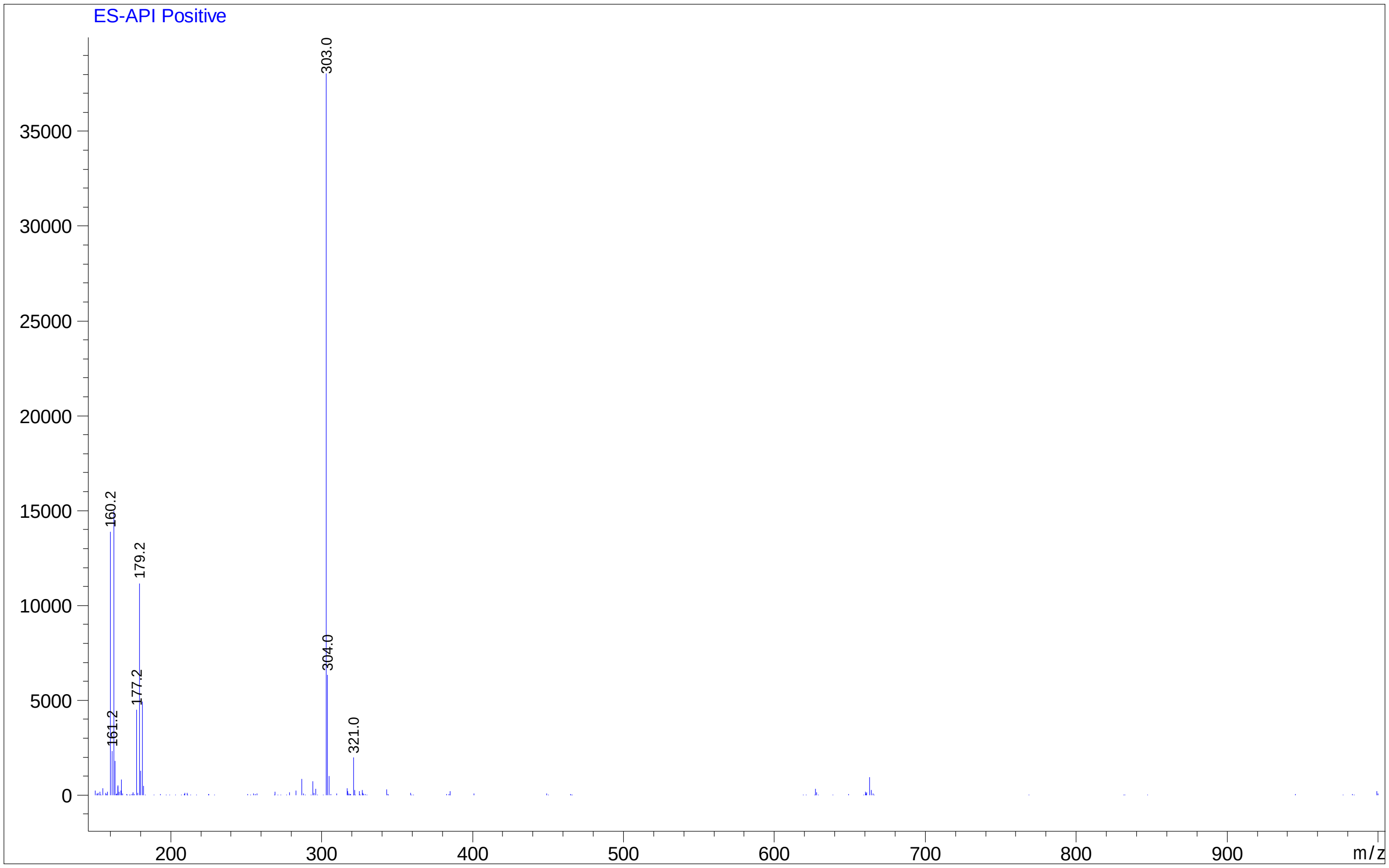

UPLC-MS-10 10/19/2023 8:49:52 AM LY_2278

Supplementary Figure 3. NMR spectra for delphinidin

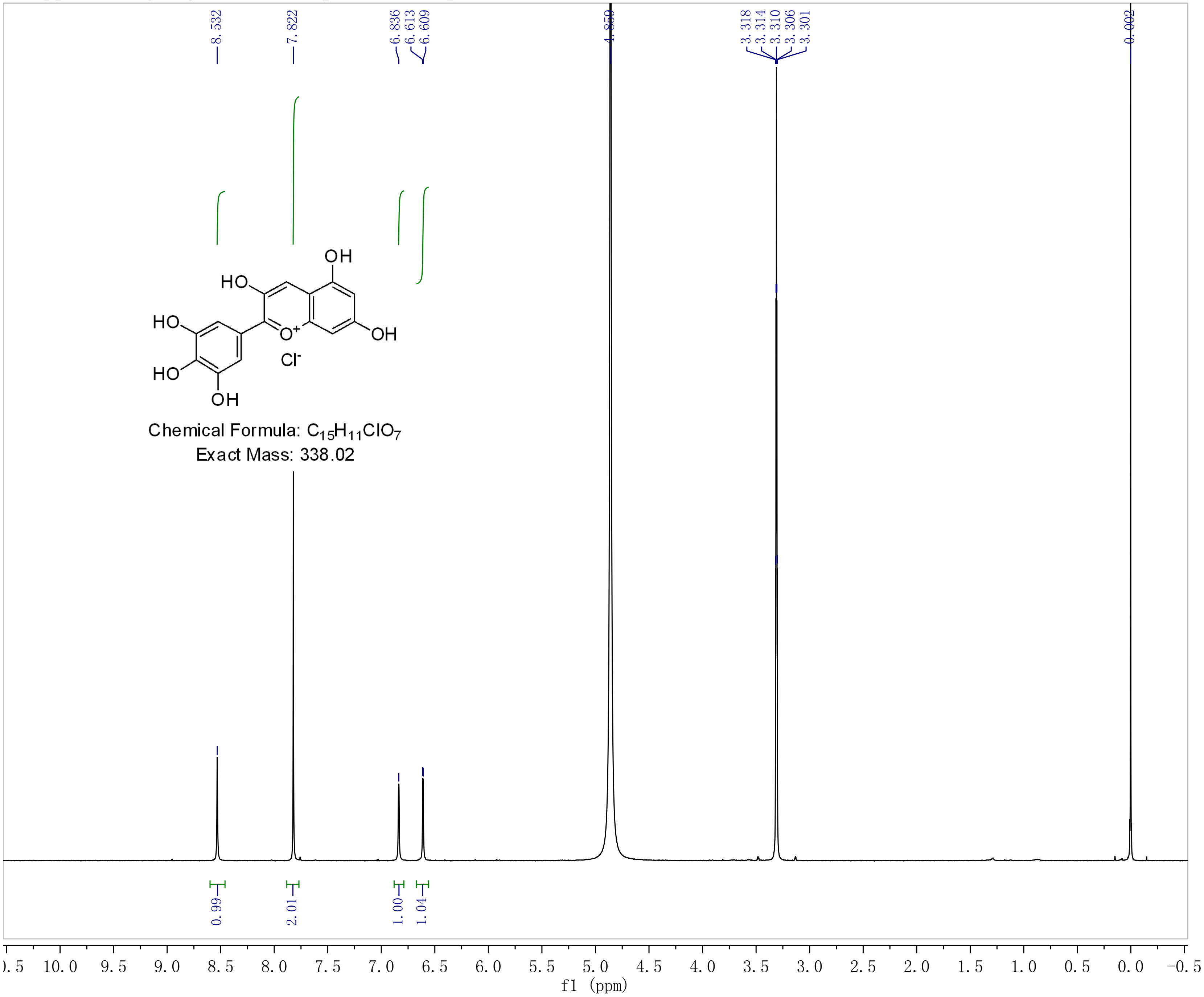

	Parameter	Value
1	Title	
2	Origin	Bruker BioSpin GmbH
3	Owner	nmrsu
4	Spectrometer	Avance
5	Solvent	MeOD
6	Temperature	298.2
7	Pulse Sequence	zg30
8	Experiment	1D
9	Number of Scans	8
10	Receiver Gain	101
11	Relaxation Delay	1.0000
12	Pulse Width	8.0000
13	Acquisition Time	3.9977
14	Acquisition Date	2023-10-18T17:09:56
15	Modification Date	2023-10-18T17:19:33
16	Spectrometer Frequency	400.13
17	Spectral Width	8196.7
18	Nucleus	1H
